# Supplementary material for: Noncanonical roles of ATG5 and membrane atg8ylation in retromer assembly and function
Source: eLife. 2025 Jan 7;13:RP100928. doi: 10.7554/eLife.100928 (PMC11706607; doi:10.7554/eLife.100928)

LOW EXPOSURE

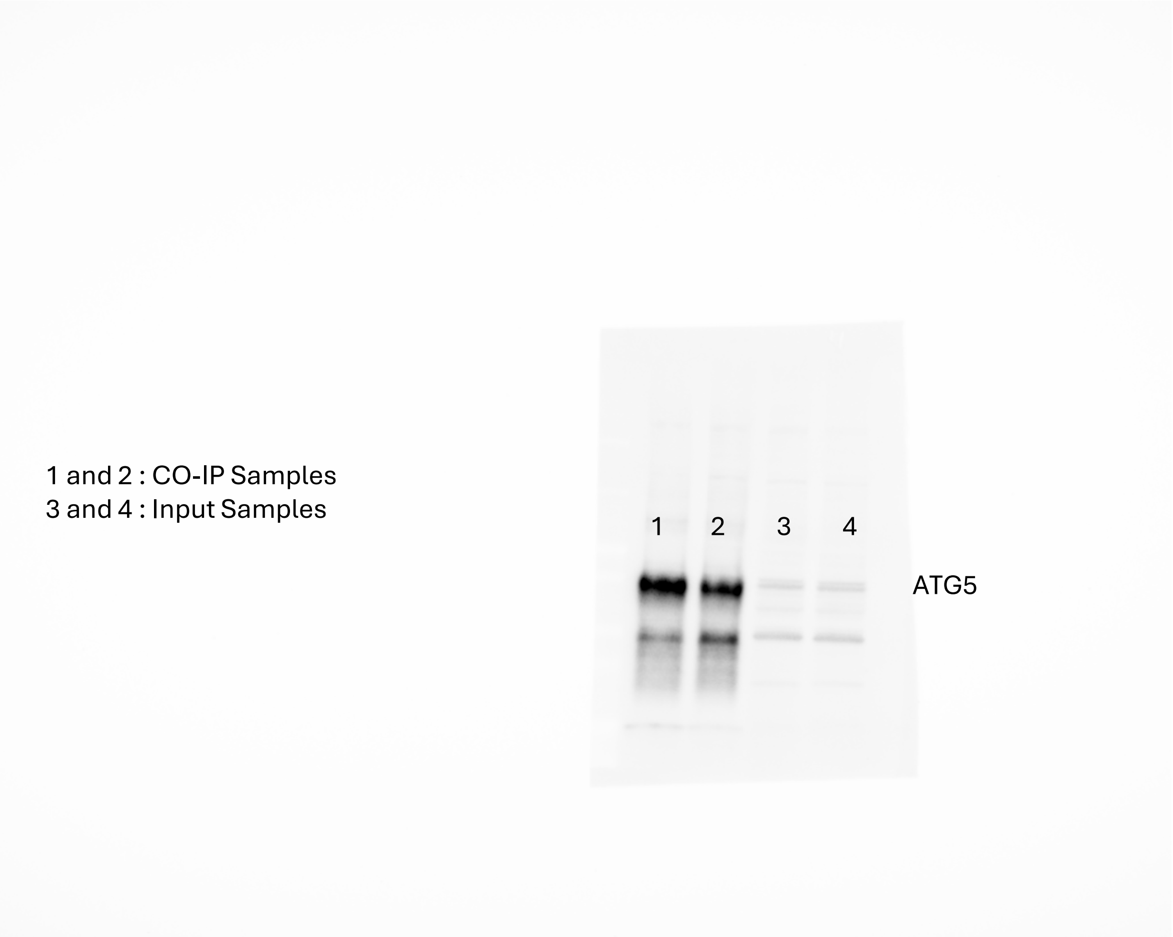

HIGH EXPOSURE

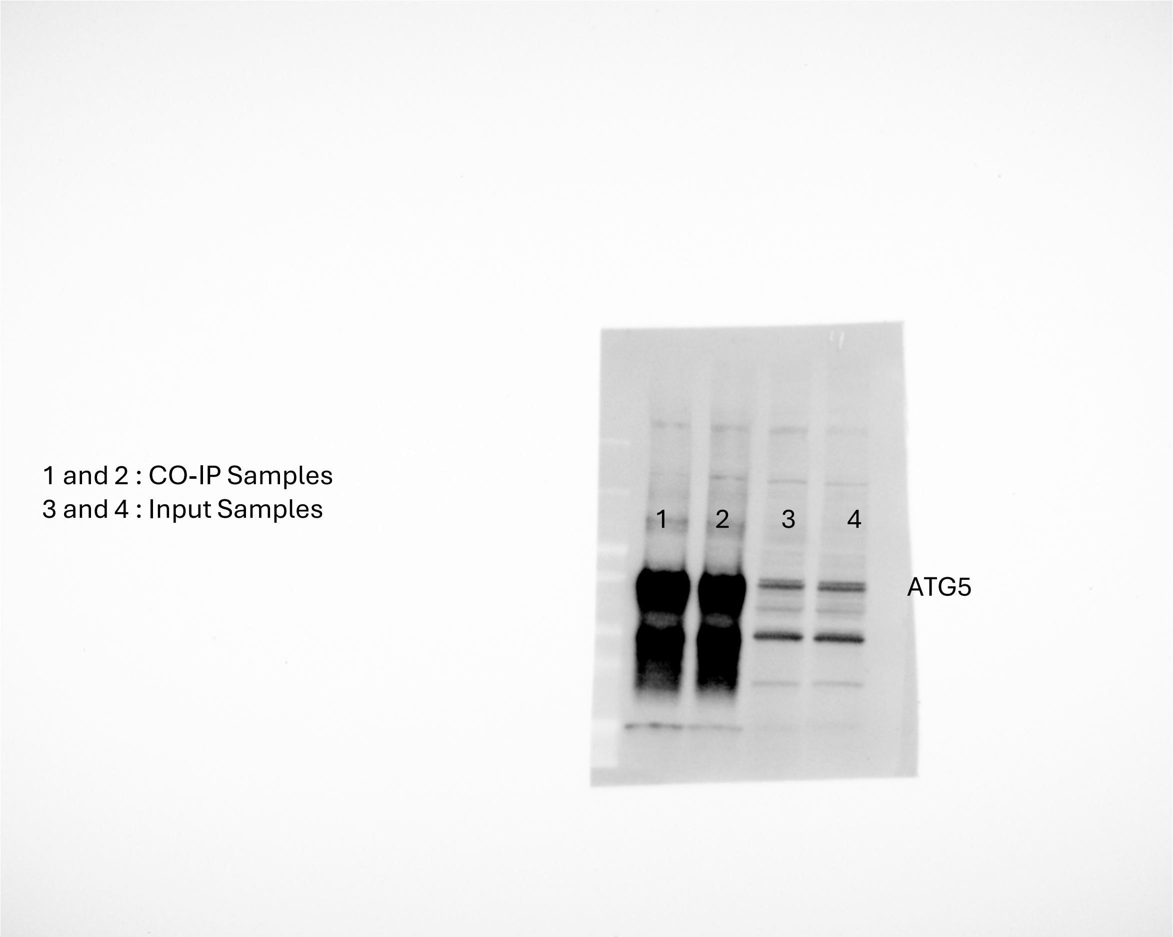

Supplement: Figure 1—source data 1. [file elife-100928-fig1-data1.zip › Figure 1 - Source data 1/Figure 1 - source data 2.2 uncropped and labelled.pdf]
