# Supplementary figures and images for: Noncanonical roles of ATG5 and membrane atg8ylation in retromer assembly and function
Source: eLife. 2025 Jan 7;13:RP100928. doi: 10.7554/eLife.100928 (PMC11706607; doi:10.7554/eLife.100928)

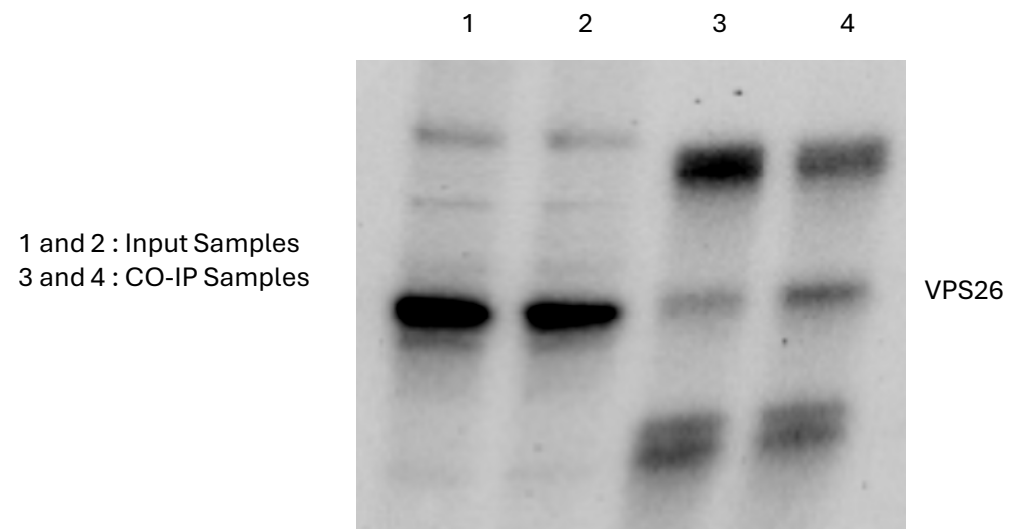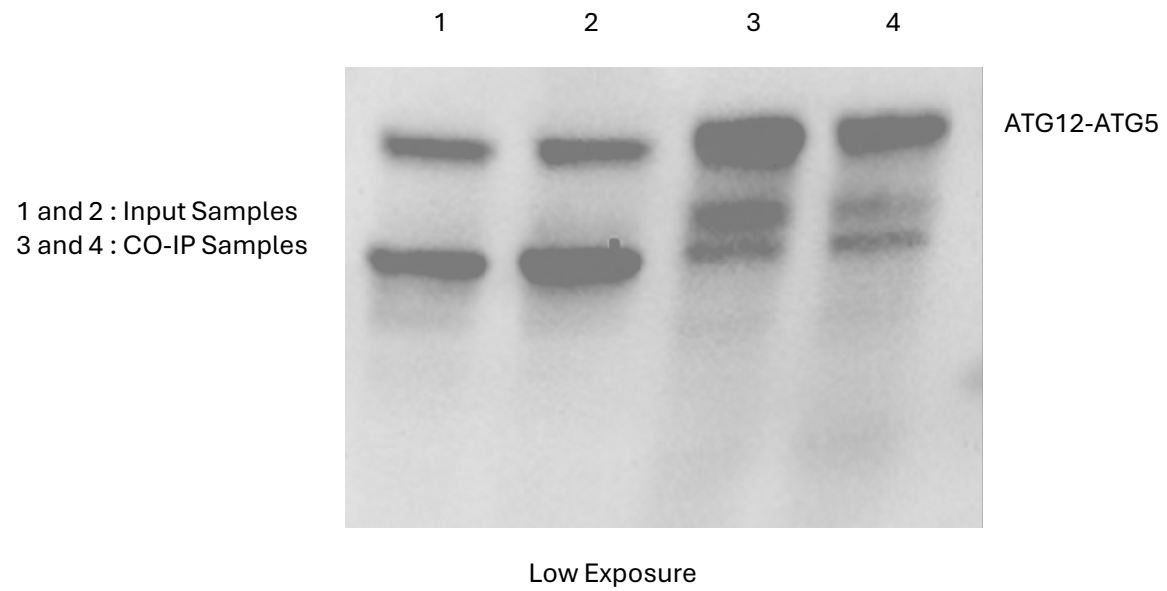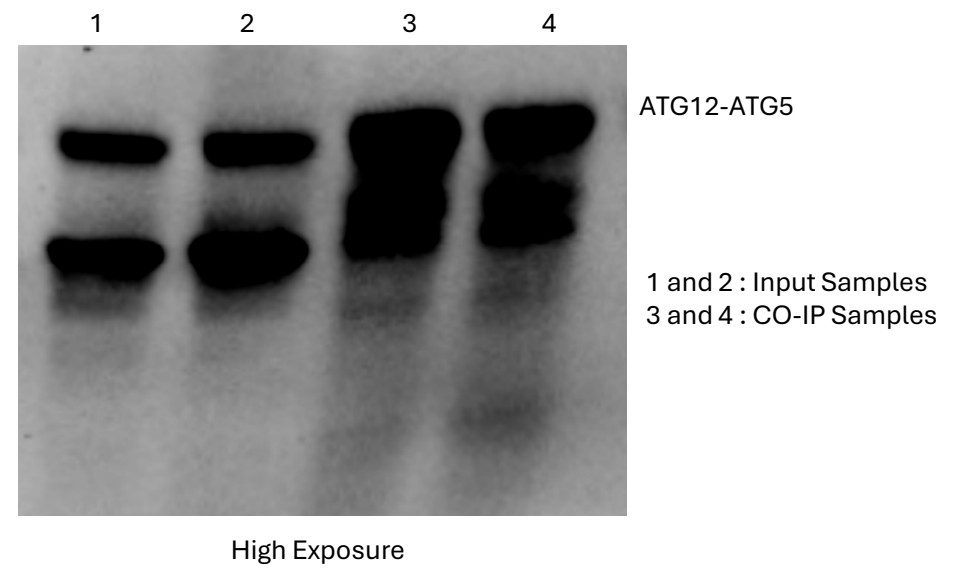

Supplement: Figure 1—source data 1. [file elife-100928-fig1-data1.zip › Figure 1 - Source data 1/Figure 1 - source data 1.1 uncropped and labelled.pdf]

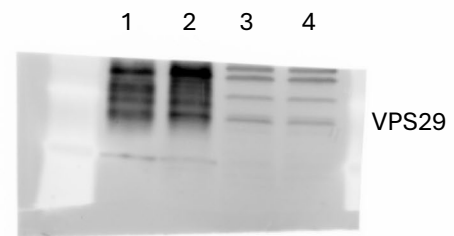

1 and 2 : CO-IP Samples  
3 and 4 : Input Samples

Supplement: Figure 1—source data 1. [file elife-100928-fig1-data1.zip › Figure 1 - Source data 1/Figure 1 - source data 2.1 uncropped and labelled.pdf]

1 and 2 : CO-IP Samples  
3 and 4 : Input Samples

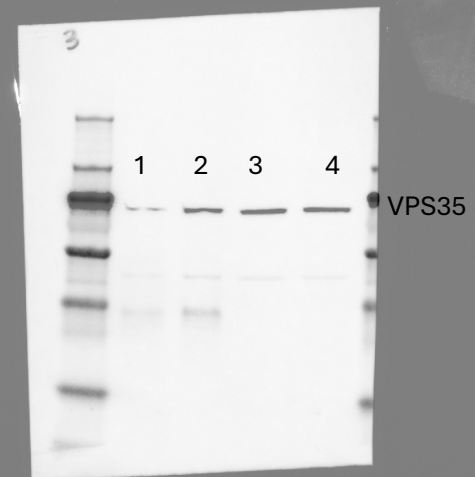

Supplement: Figure 1—source data 1. [file elife-100928-fig1-data1.zip › Figure 1 - Source data 1/Figure 1 - source data 3.1 uncropped and labelled.pdf]

## LOW EXPOSURE

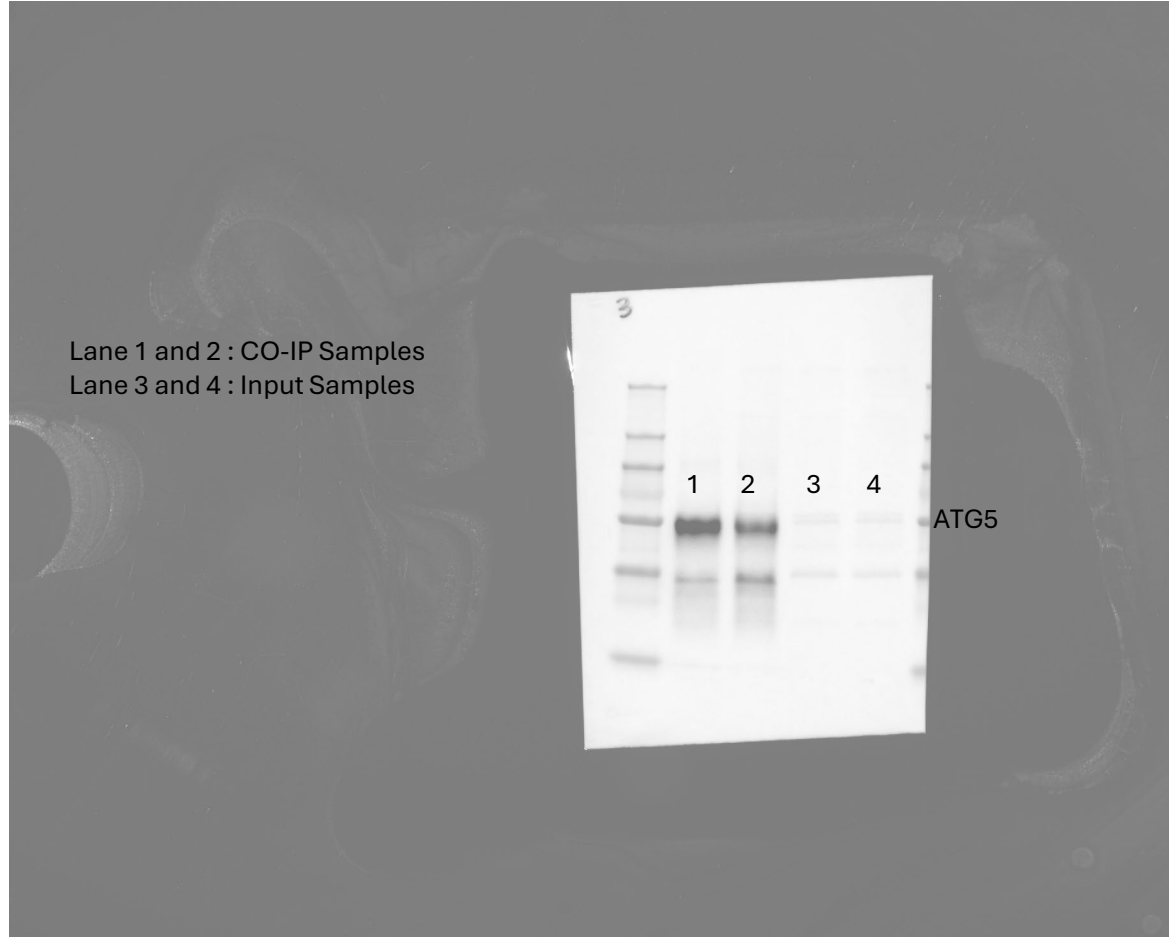

## HIGH EXPOSURE

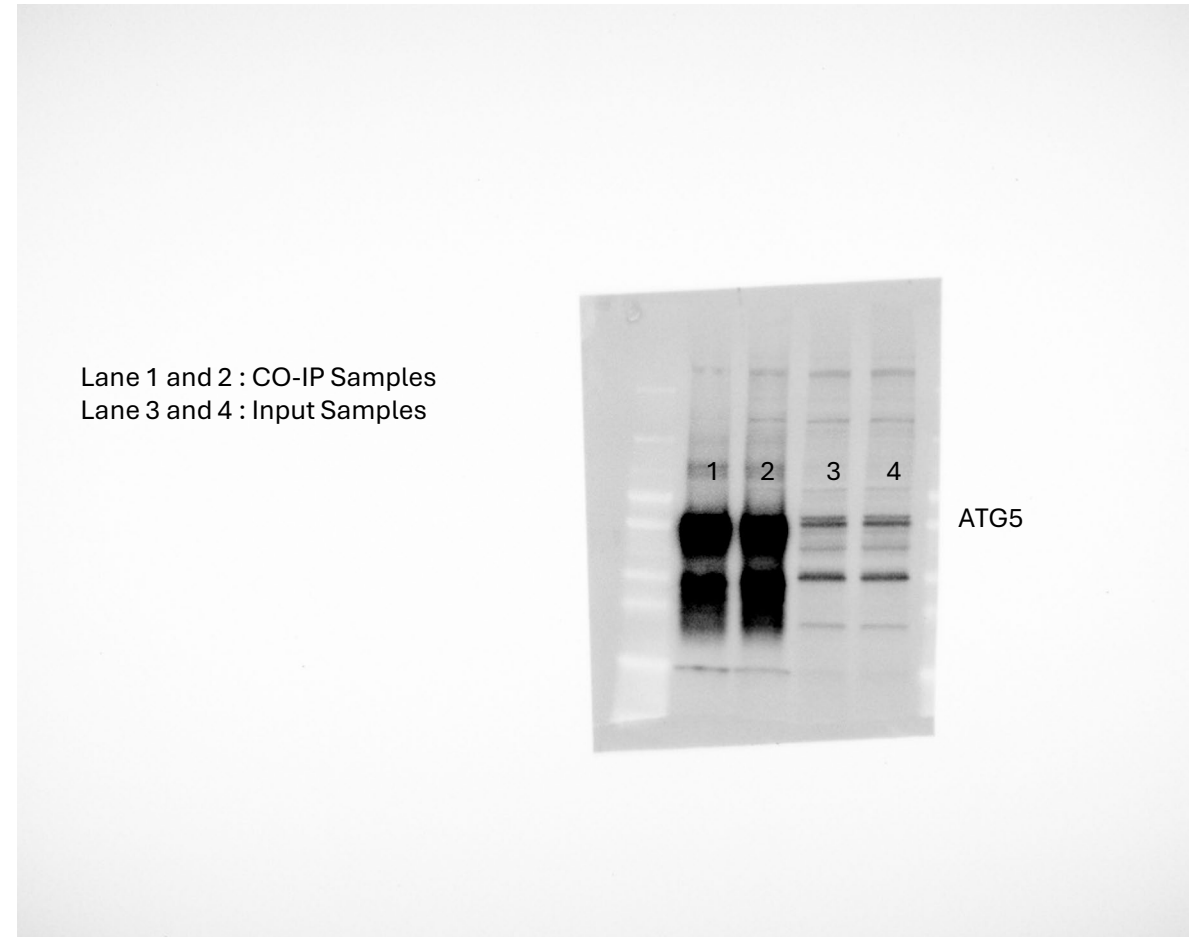

Supplement: Figure 1—source data 1. [file elife-100928-fig1-data1.zip › Figure 1 - Source data 1/Figure 1 - source data 3.2 uncropped and labelled.pdf]

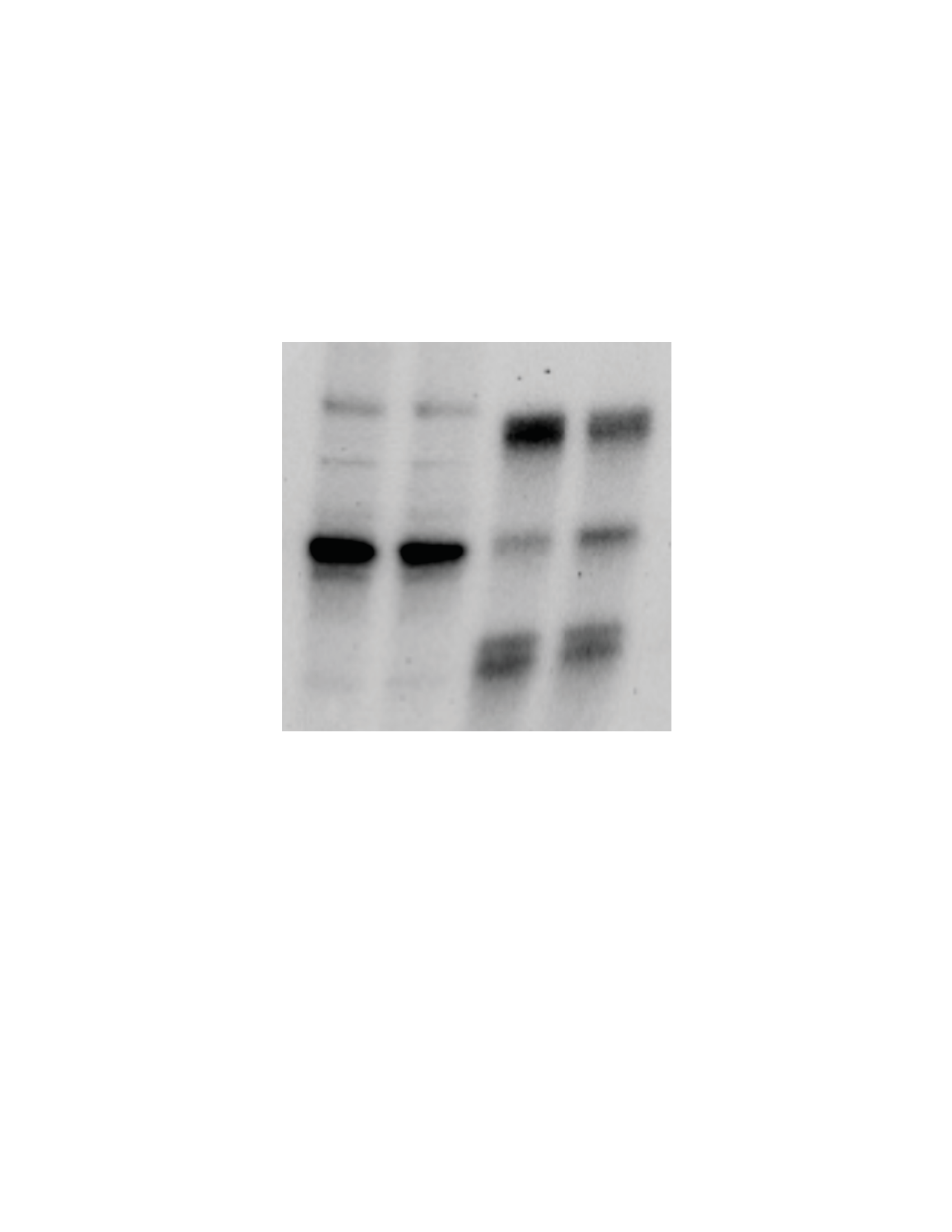

Supplement: Figure 1—source data 2. [file elife-100928-fig1-data2.zip › Figure 1 - Source data 2/1.1.tiff]

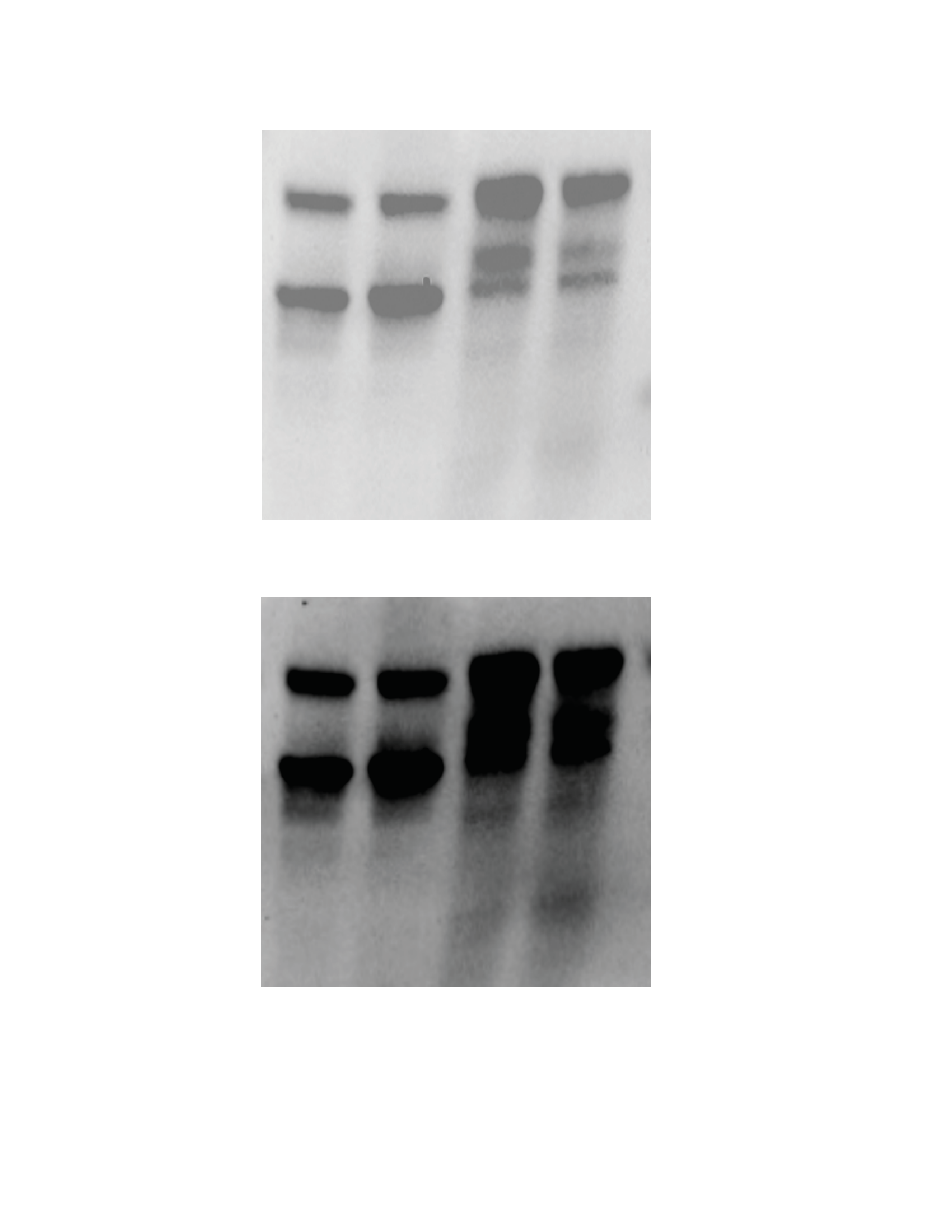

Supplement: Figure 1—source data 2. [file elife-100928-fig1-data2.zip › Figure 1 - Source data 2/1.2.tiff]

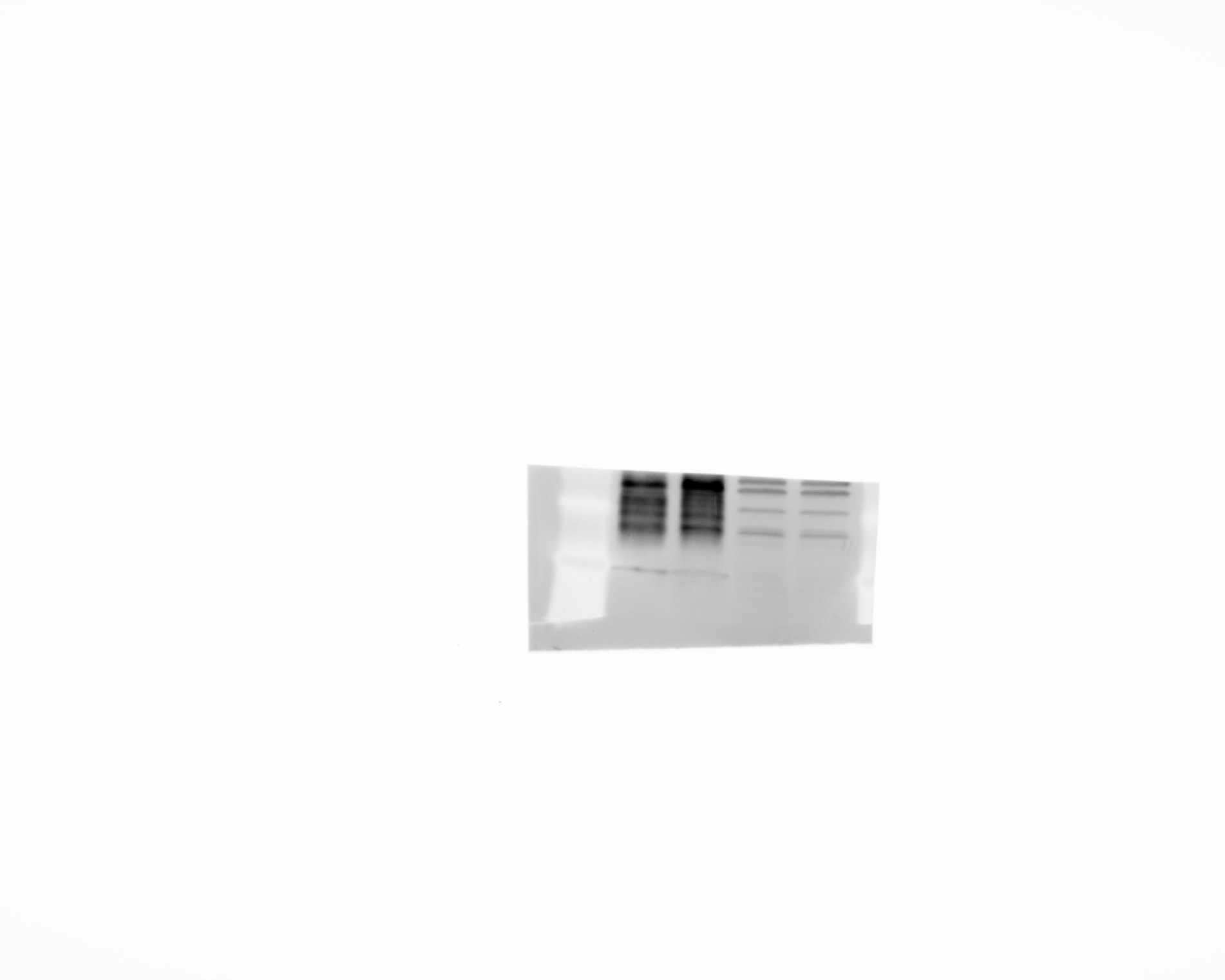

Supplement: Figure 1—source data 2. [file elife-100928-fig1-data2.zip › Figure 1 - Source data 2/2.1 2023-11-08 18h03m52s Deretic Lab(IRDye 680RD).tif]

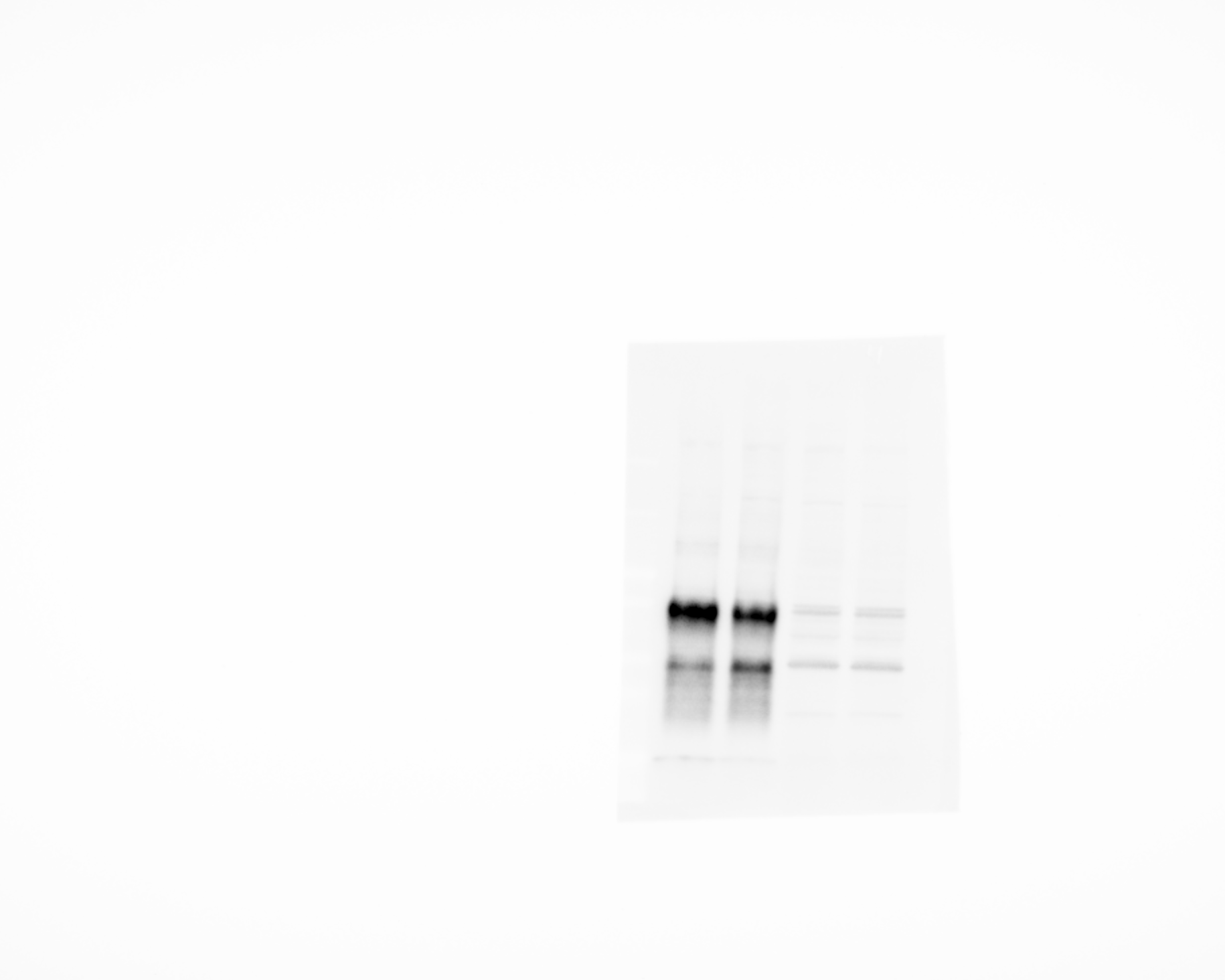

Supplement: Figure 1—source data 2. [file elife-100928-fig1-data2.zip › Figure 1 - Source data 2/2.2 2023-10-05 18h47m00s Deretic Lab(IRDye 800CW).tif]

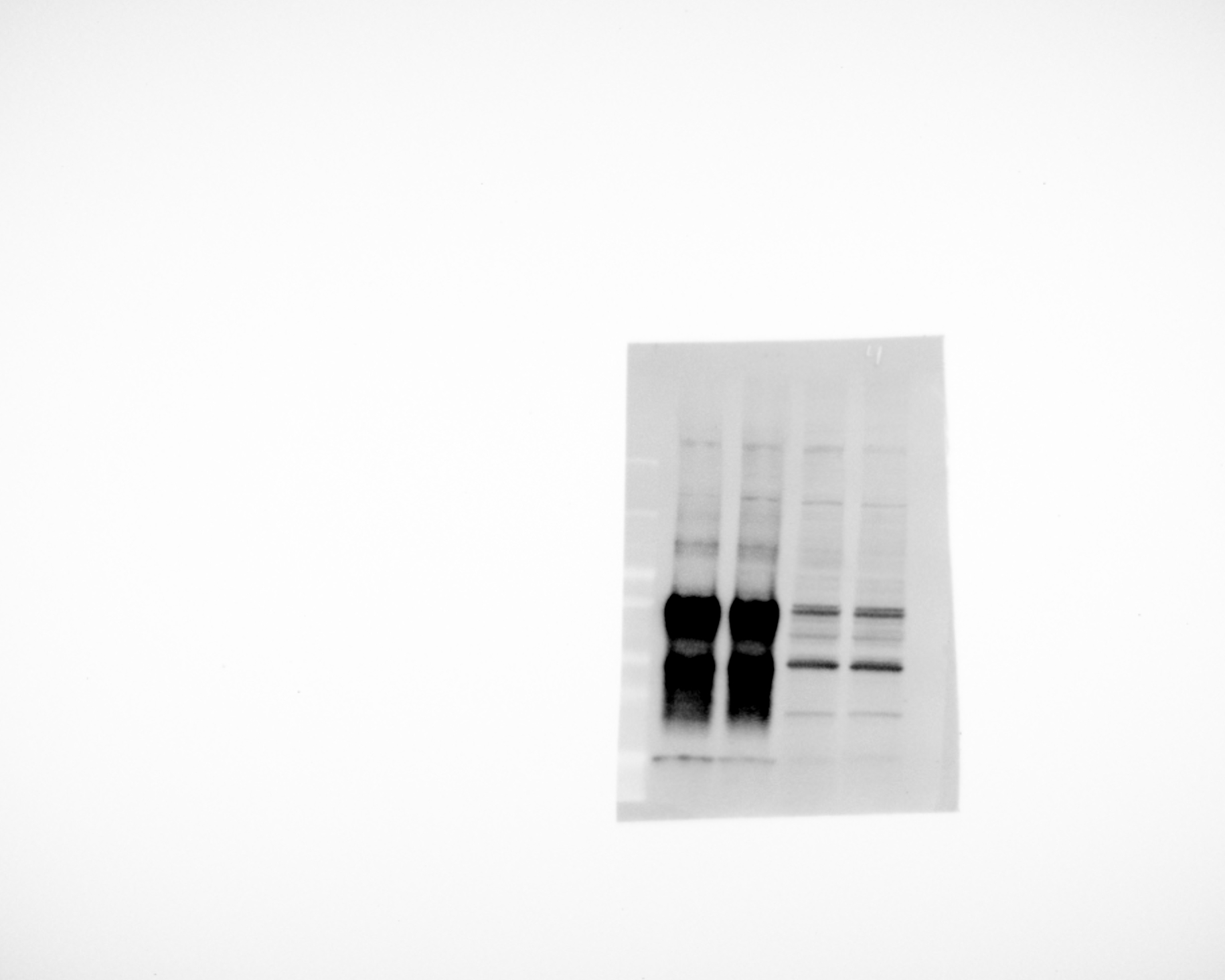

Supplement: Figure 1—source data 2. [file elife-100928-fig1-data2.zip › Figure 1 - Source data 2/2.2. 2023-10-05 18h47m22s Deretic Lab(IRDye 800CW).jpg]

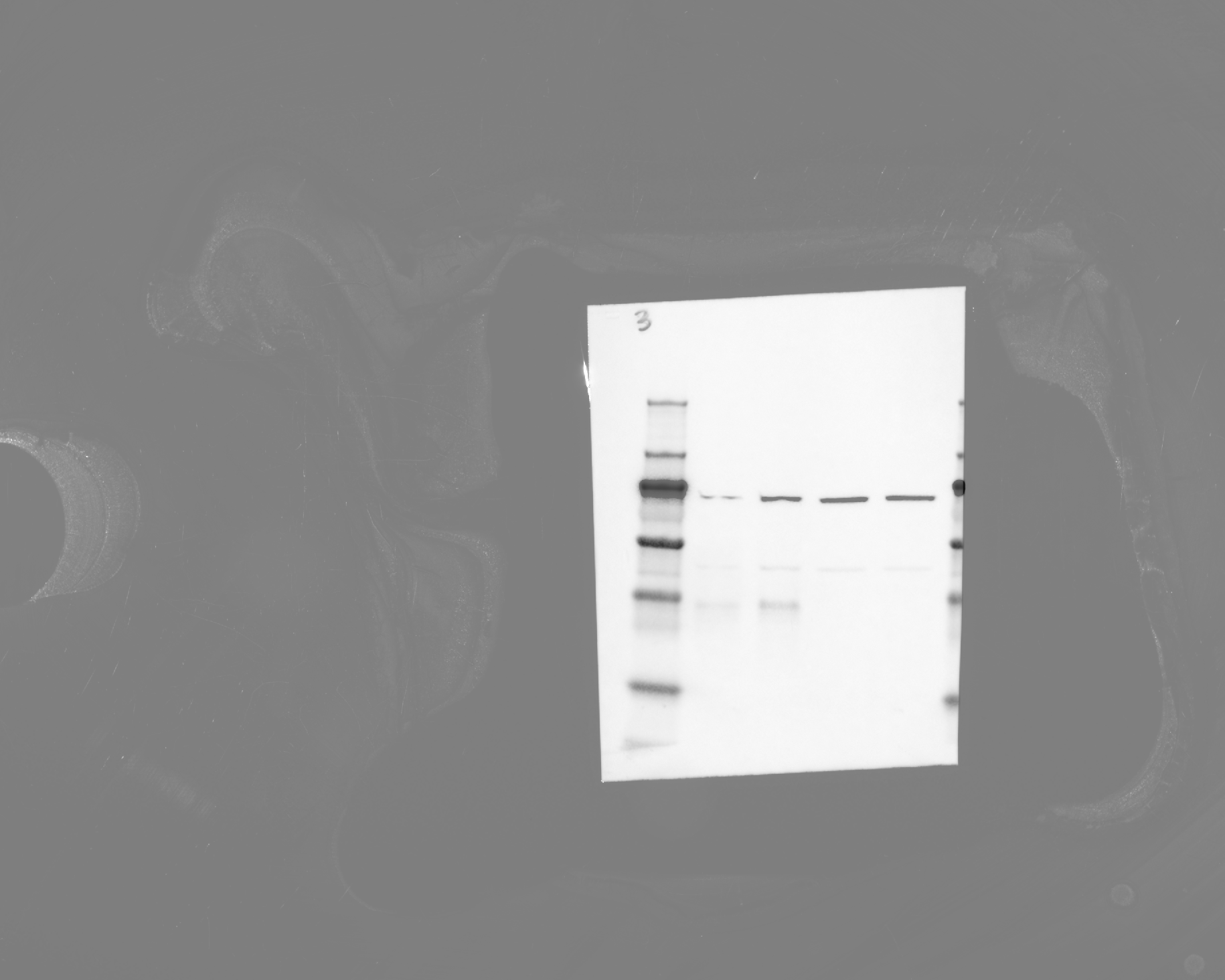

Supplement: Figure 1—source data 2. [file elife-100928-fig1-data2.zip › Figure 1 - Source data 2/3.1 2023-10-05 18h46m12s Deretic Lab(Composite).tif]

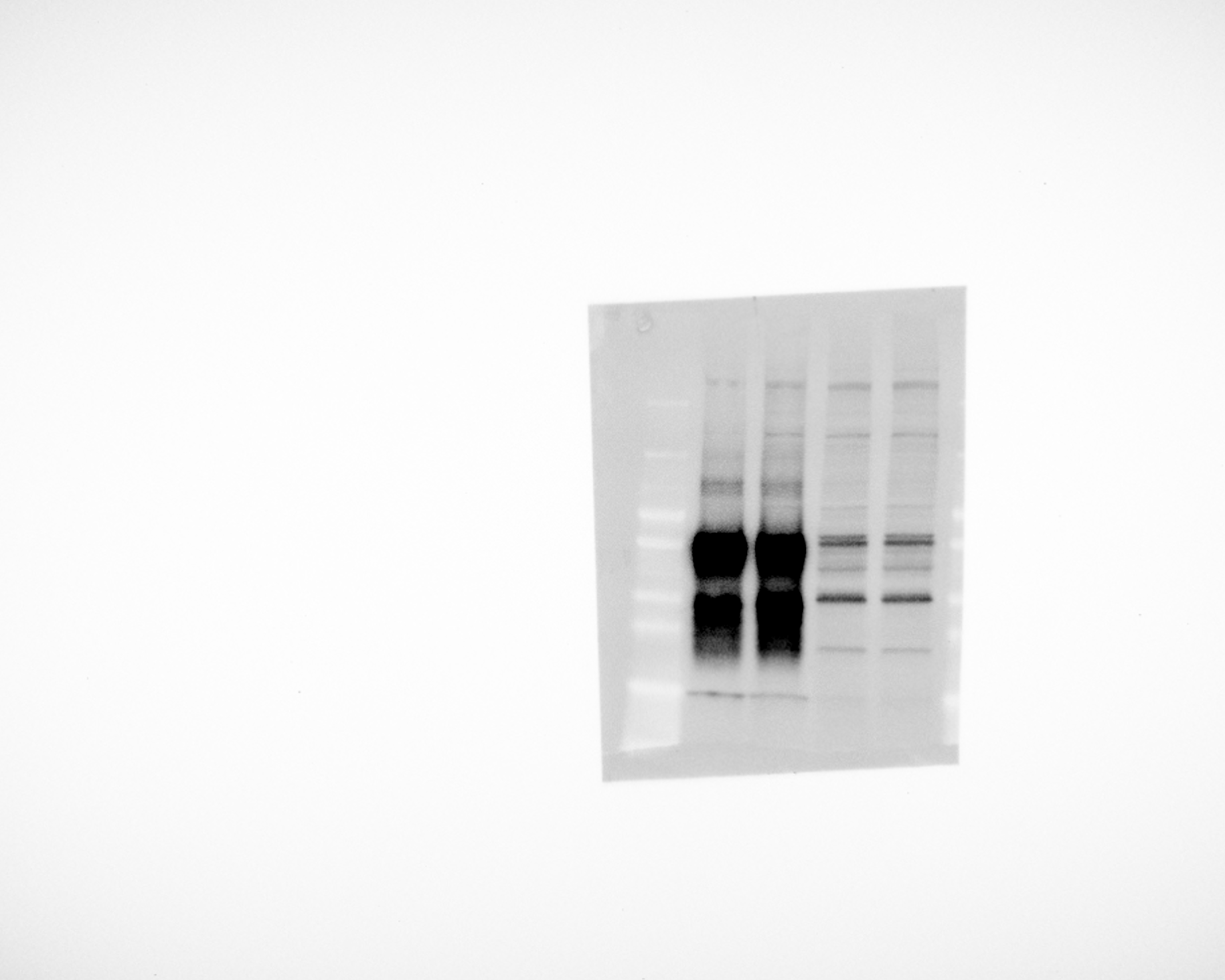

Supplement: Figure 1—source data 2. [file elife-100928-fig1-data2.zip › Figure 1 - Source data 2/3.2 2023-10-05 18h45m47s Deretic Lab(IRDye 800CW).tif]

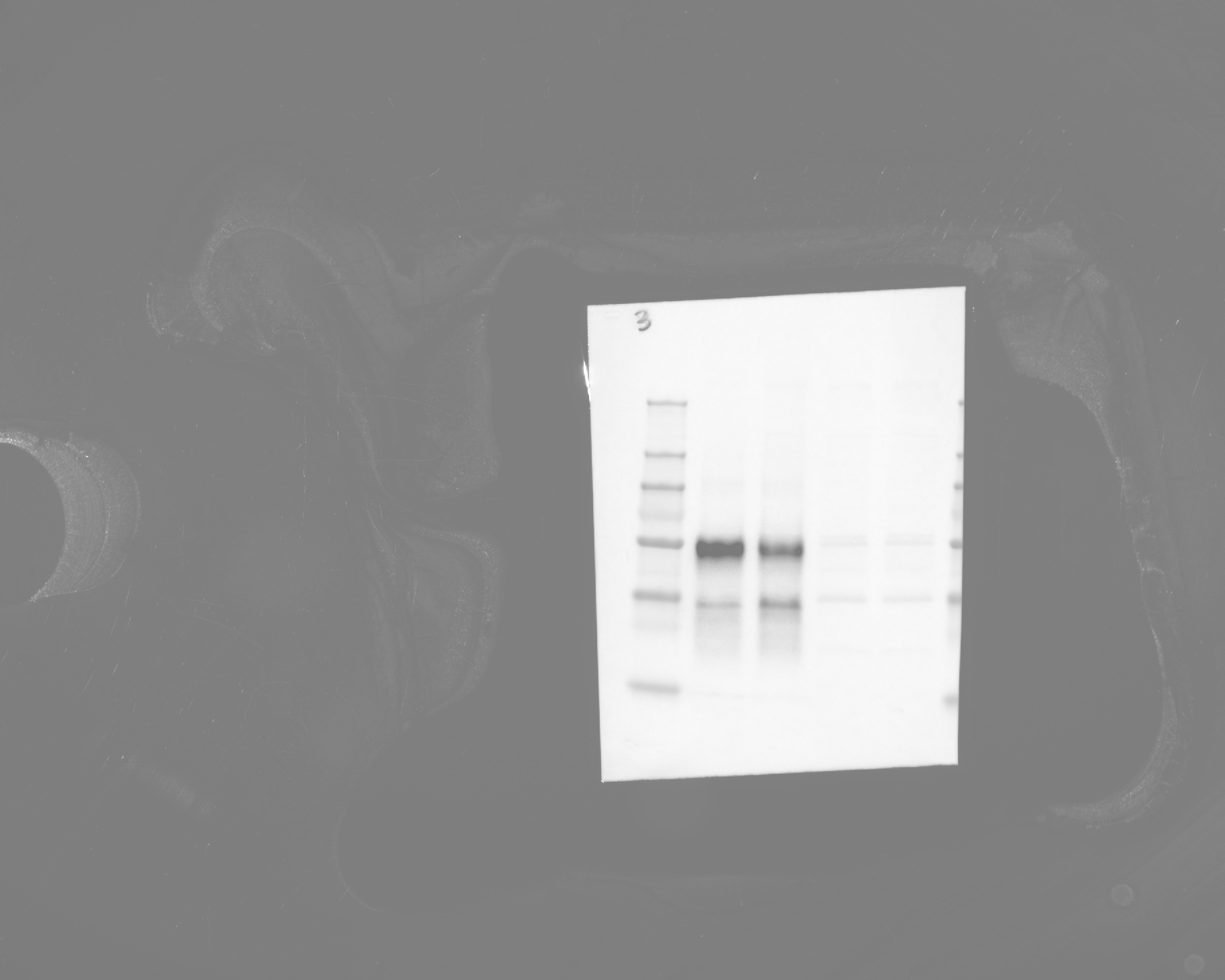

Supplement: Figure 1—source data 2. [file elife-100928-fig1-data2.zip › Figure 1 - Source data 2/3.2 2023-10-05 18h46m02s Deretic Lab(Composite).jpg]

1 and 2 : CO-IP Samples  
3 and 4 : Input Samples

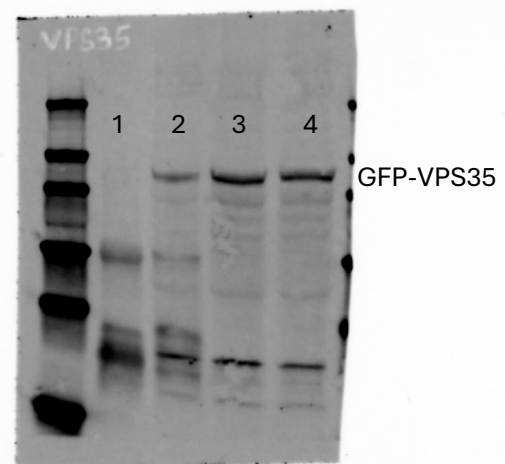

Supplement: Figure 3—source data 1. [file elife-100928-fig3-data1.zip › Figure 3 - Source data 1/Figure 3 - source data 1.1 uncropped and labelled.pdf]

1 and 2 : CO-IP Samples  
3 and 4 : Input Samples

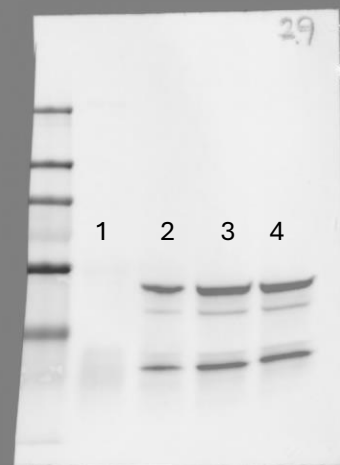

YFP-VPS29

Supplement: Figure 3—source data 1. [file elife-100928-fig3-data1.zip › Figure 3 - Source data 1/Figure 3 - source data 1.2 uncropped and labelled.pdf]

1 and 2 : CO-IP Samples  
3 and 4 : Input Samples

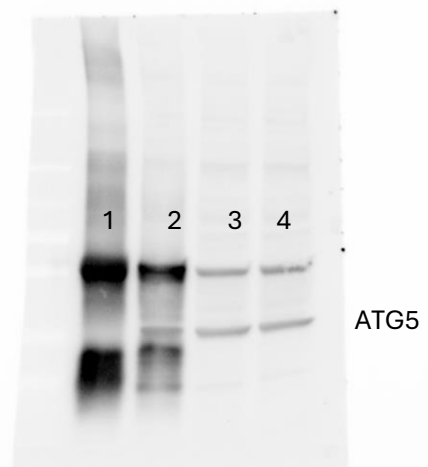

Supplement: Figure 3—source data 1. [file elife-100928-fig3-data1.zip › Figure 3 - Source data 1/Figure 3 - source data 1.3 uncropped and labelled.pdf]

1 to 3 : CO-IP Samples  
4 to 6 : Input Samples

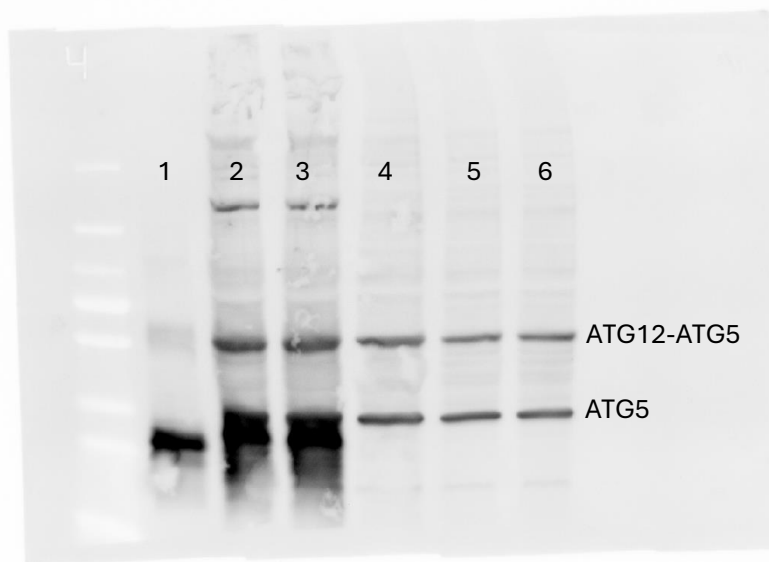

Supplement: Figure 3—source data 1. [file elife-100928-fig3-data1.zip › Figure 3 - Source data 1/Figure 3 - source data 2.1 uncropped and labelled.pdf]

1 to 3 : CO-IP Samples  
4 to 6 : Input Samples

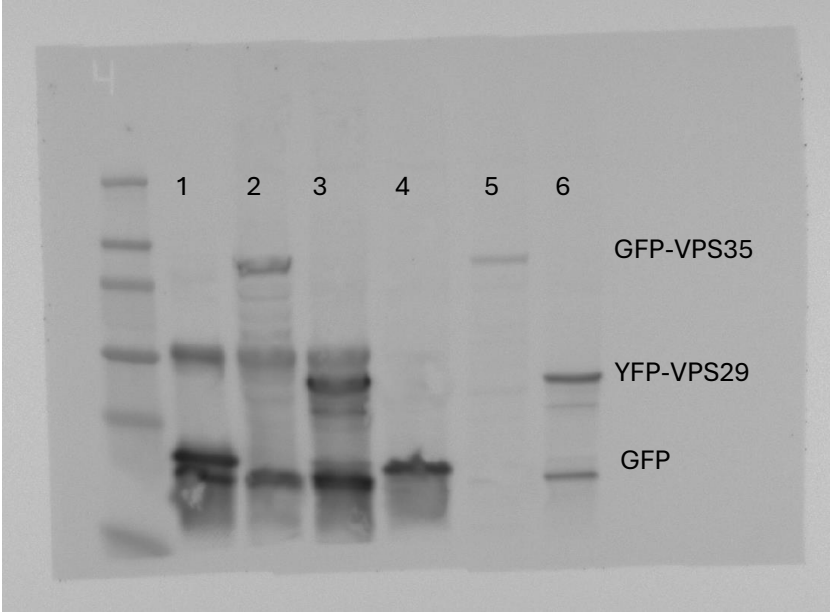

Supplement: Figure 3—source data 1. [file elife-100928-fig3-data1.zip › Figure 3 - Source data 1/Figure 3 - source data 2.2 uncropped and labelled.pdf]

1 to 4 : Lyso-IP Samples  
5 to 8 : Input Samples

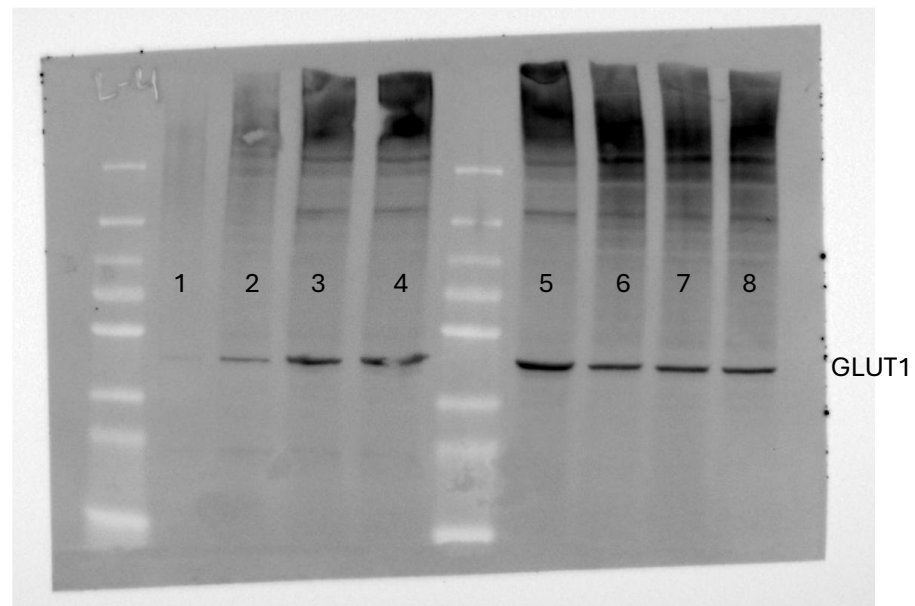

Supplement: Figure 3—source data 1. [file elife-100928-fig3-data1.zip › Figure 3 - Source data 1/Figure 3 - source data 3.1 uncropped and labelled.pdf]

1 to 4 : Lyso-IP Samples  
5 to 8 : Input Samples

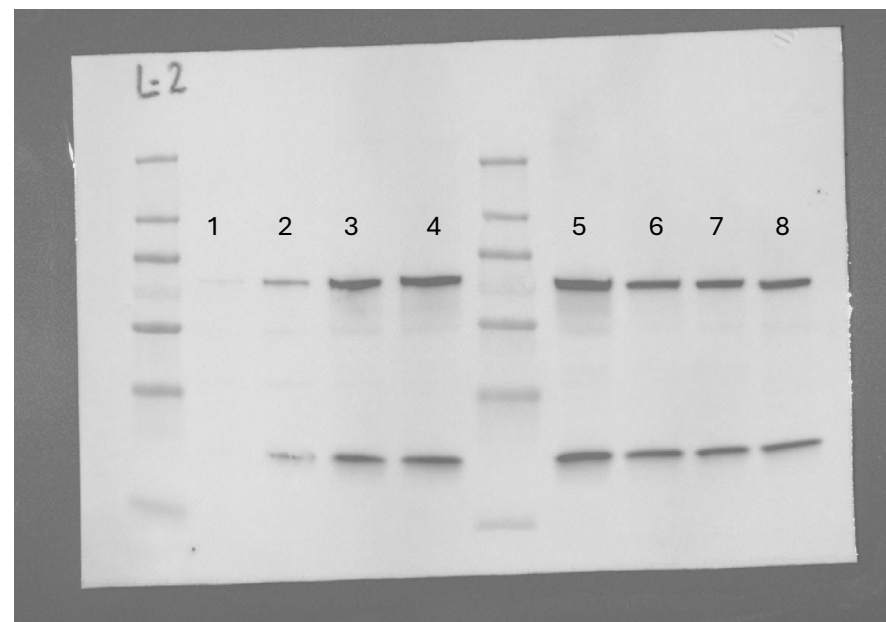

SNX27

Supplement: Figure 3—source data 1. [file elife-100928-fig3-data1.zip › Figure 3 - Source data 1/Figure 3 - source data 3.2 uncropped and labelled.pdf]

1 to 4 : Lyso-IP Samples  
5 to 8 : Input Samples

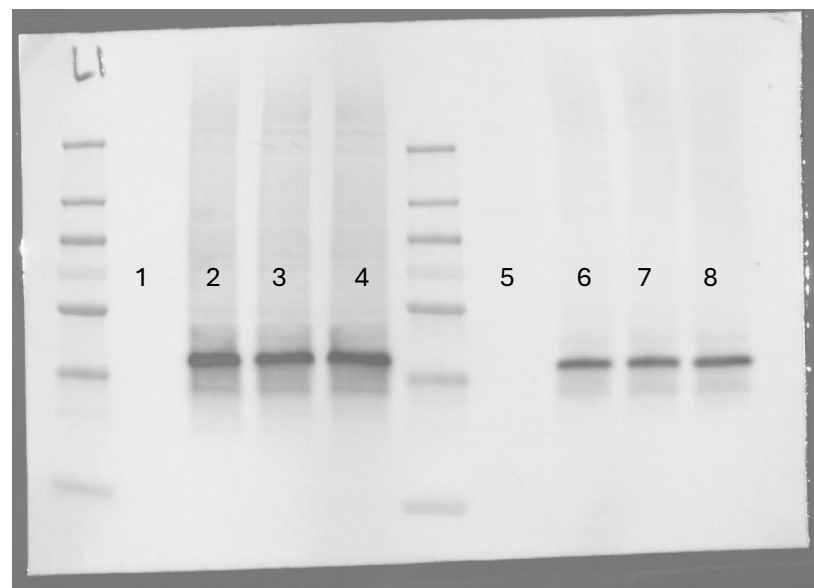

TMEM192-3xHA

Supplement: Figure 3—source data 1. [file elife-100928-fig3-data1.zip › Figure 3 - Source data 1/Figure 3 - source data 3.3 uncropped and labelled.pdf]

1 to 4 : Lyso-IP Samples  
5 to 8 : Input Samples

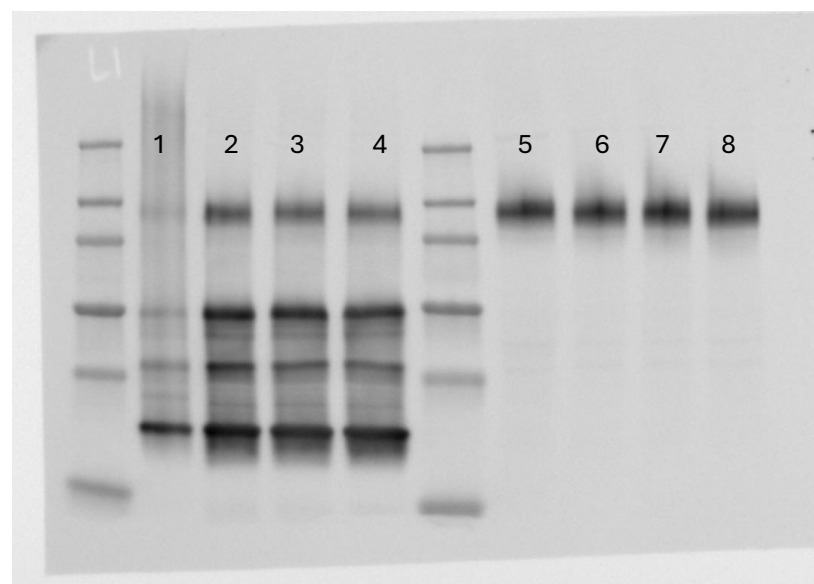

LAMP2

Supplement: Figure 3—source data 1. [file elife-100928-fig3-data1.zip › Figure 3 - Source data 1/Figure 3 - source data 3.4 uncropped and labelled.pdf]

1 to 4 : Lyso-IP Samples  
5 to 8 : Input Samples

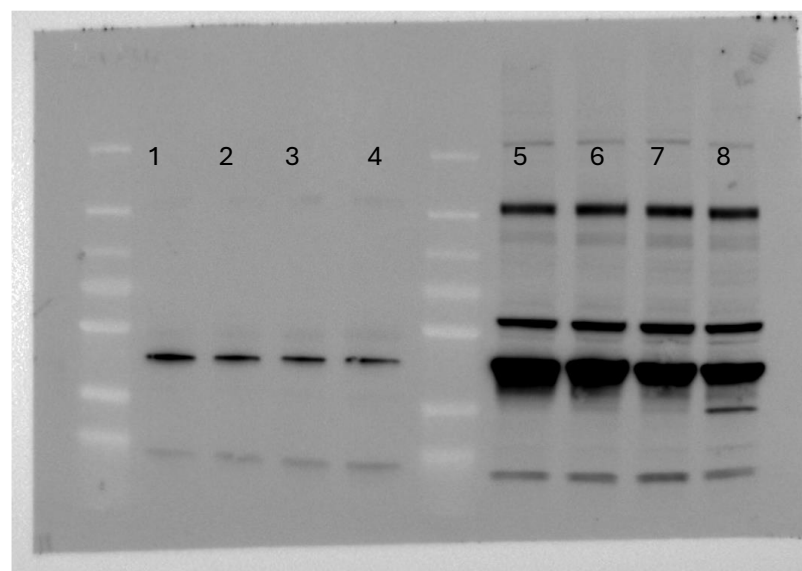

GM130 (Golgi)

PDI (ER)

Supplement: Figure 3—source data 1. [file elife-100928-fig3-data1.zip › Figure 3 - Source data 1/Figure 3 - source data 3.5 uncropped and labelled.pdf]

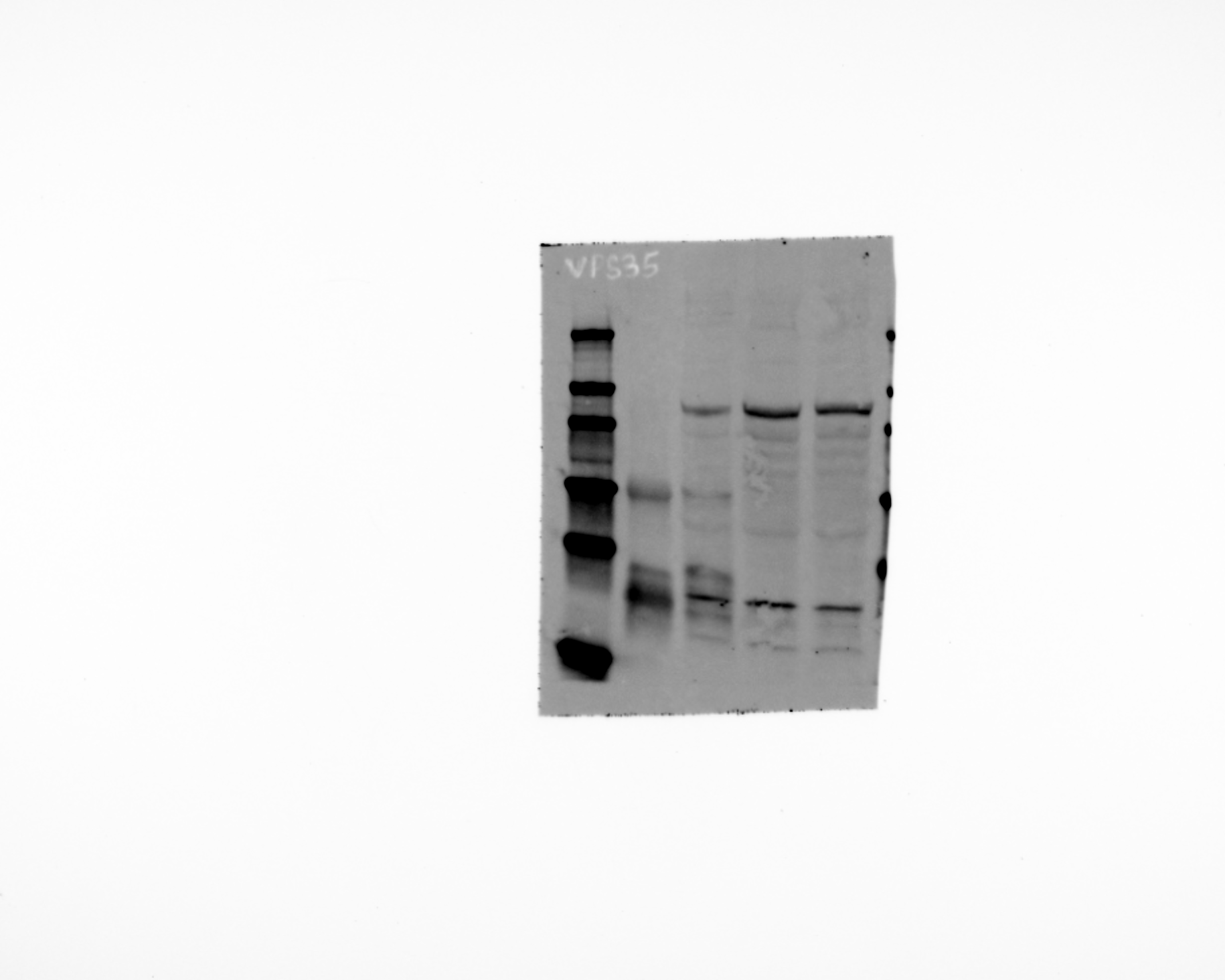

Supplement: Figure 3—source data 2. [file elife-100928-fig3-data2.zip › Figure 3 - Source data 2/1.1 2023-08-24 17h39m46s Deretic Lab(IRDye 680RD).tif]

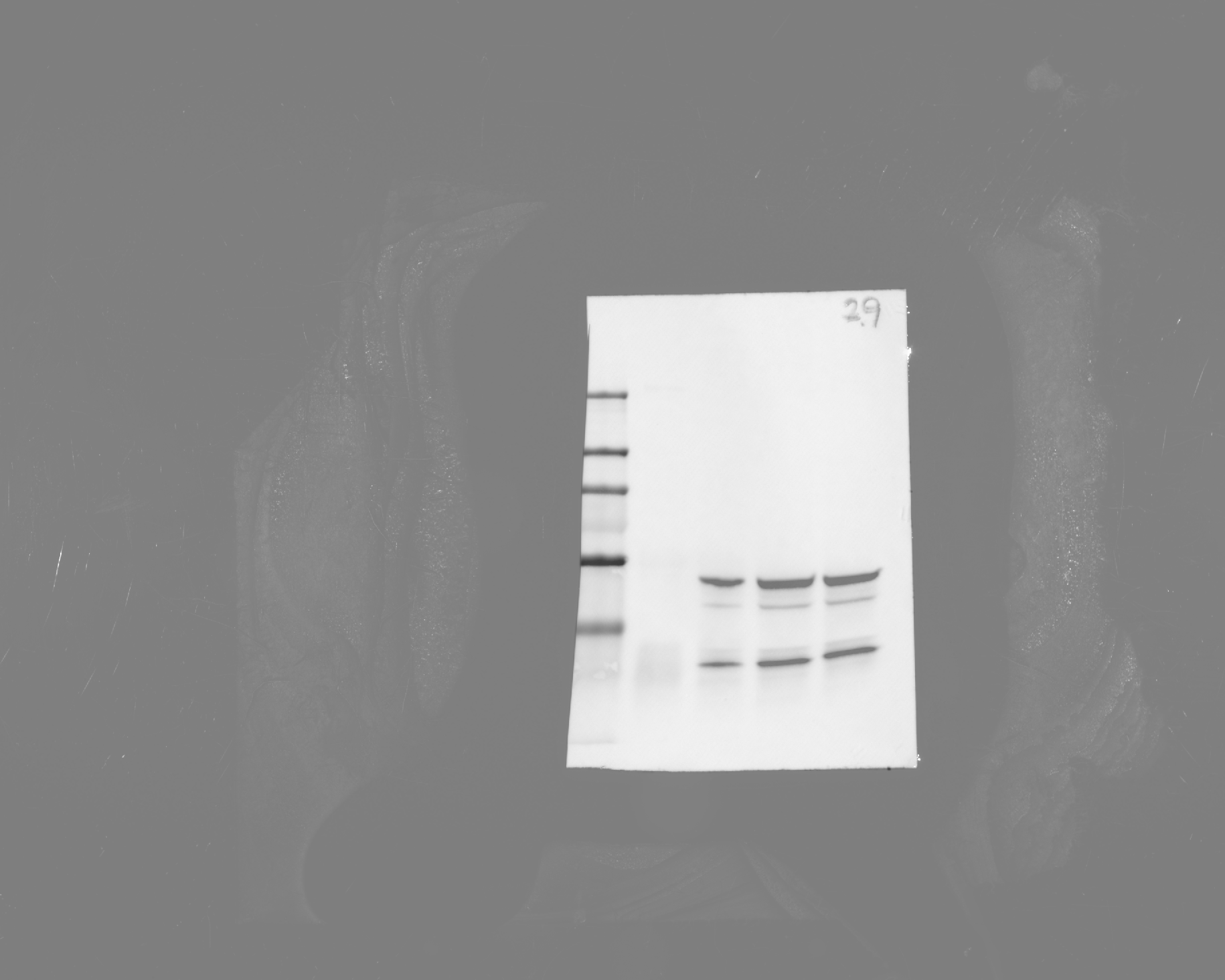

Supplement: Figure 3—source data 2. [file elife-100928-fig3-data2.zip › Figure 3 - Source data 2/1.2 2023-08-24 17h49m17s Deretic Lab(Composite).tif]

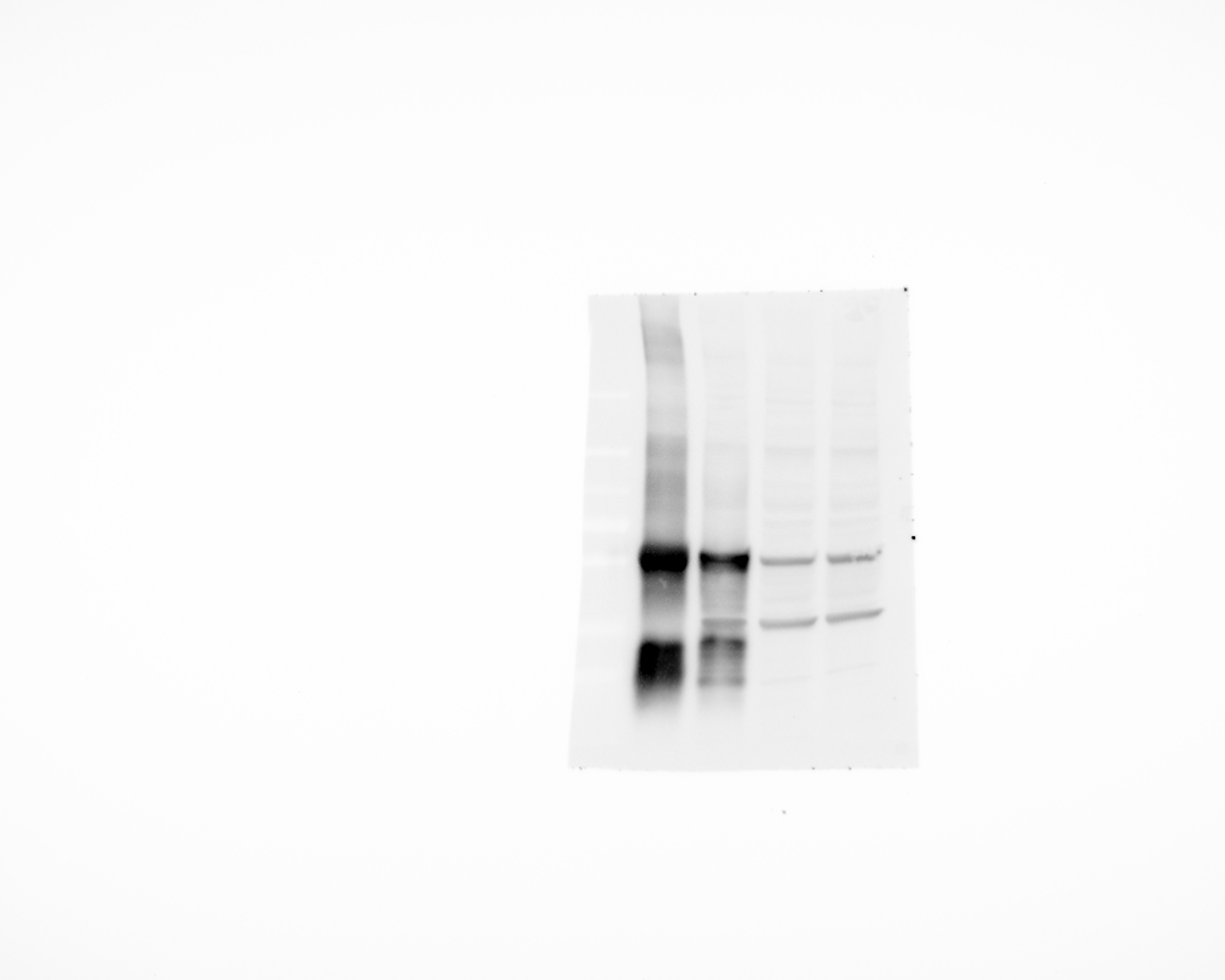

Supplement: Figure 3—source data 2. [file elife-100928-fig3-data2.zip › Figure 3 - Source data 2/1.3 2023-08-24 17h48m59s Deretic Lab(IRDye 800CW).tif]

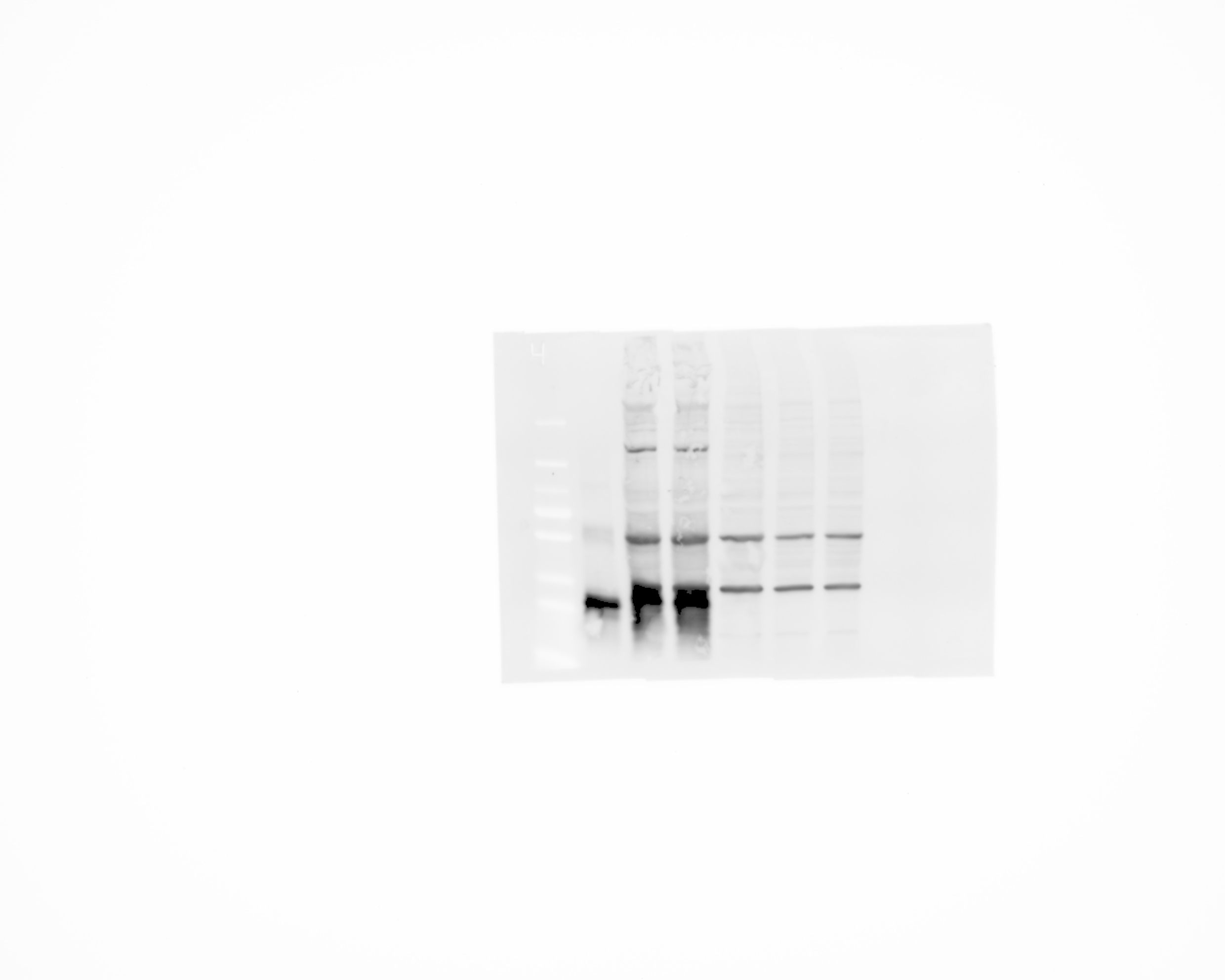

Supplement: Figure 3—source data 2. [file elife-100928-fig3-data2.zip › Figure 3 - Source data 2/2.1 2023-07-06 16h52m51s Deretic Lab(IRDye 800CW).tif]

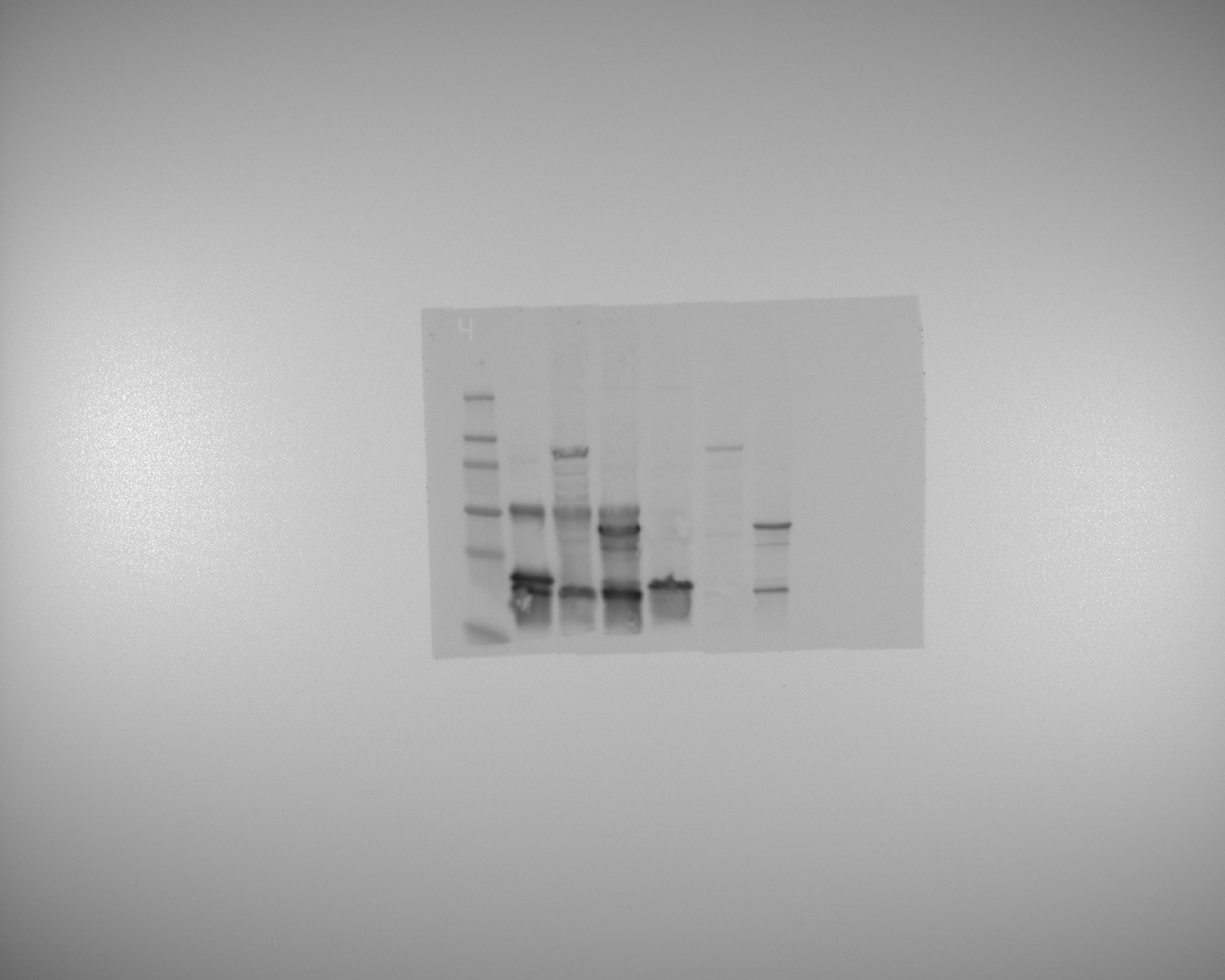

Supplement: Figure 3—source data 2. [file elife-100928-fig3-data2.zip › Figure 3 - Source data 2/2.2 2023-07-06 18h03m10s Deretic Lab(IRDye 680RD).tif]

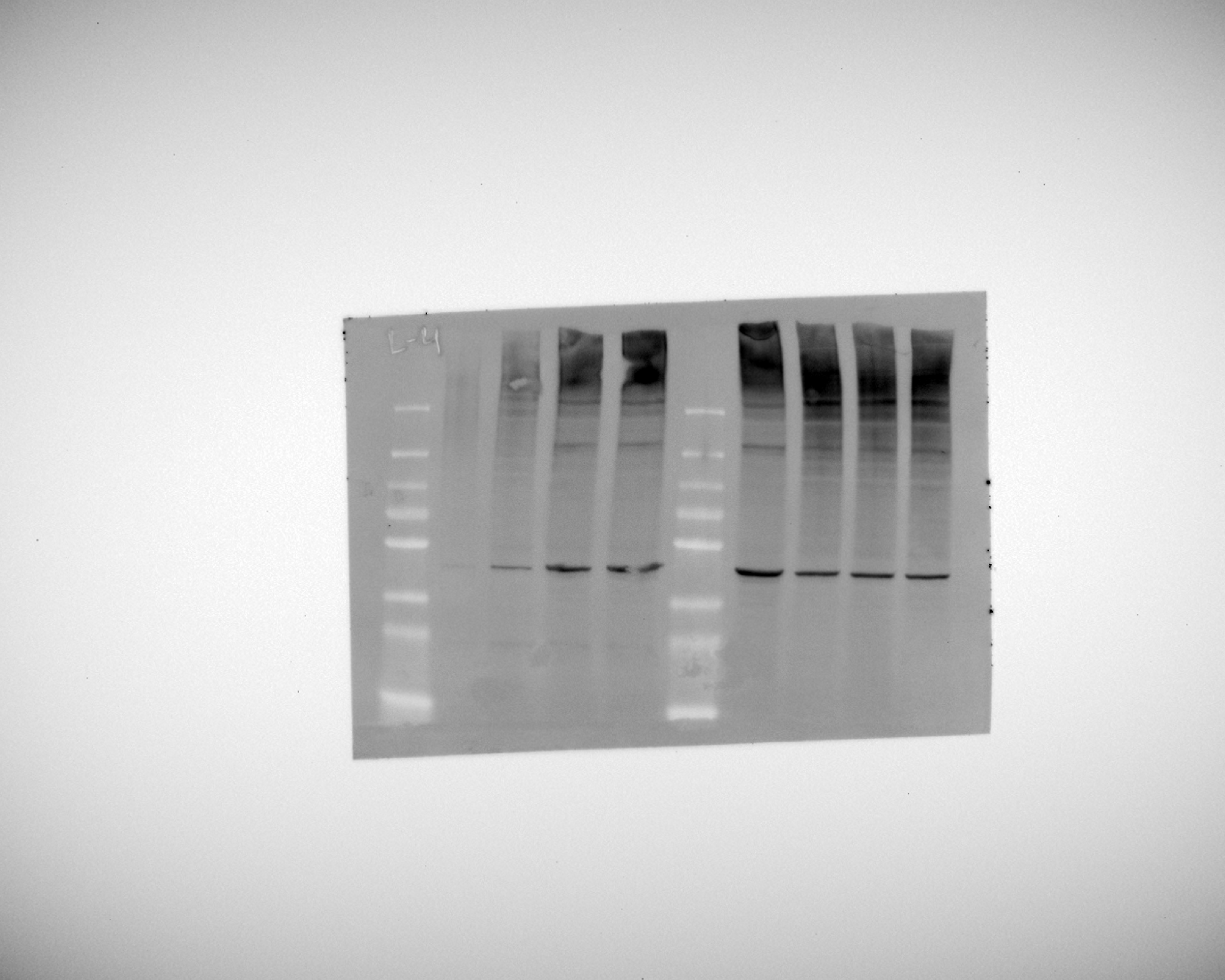

Supplement: Figure 3—source data 2. [file elife-100928-fig3-data2.zip › Figure 3 - Source data 2/3.1 D.Deretic 2024-04-04 16h59m03s(IRDye 800CW).tif]

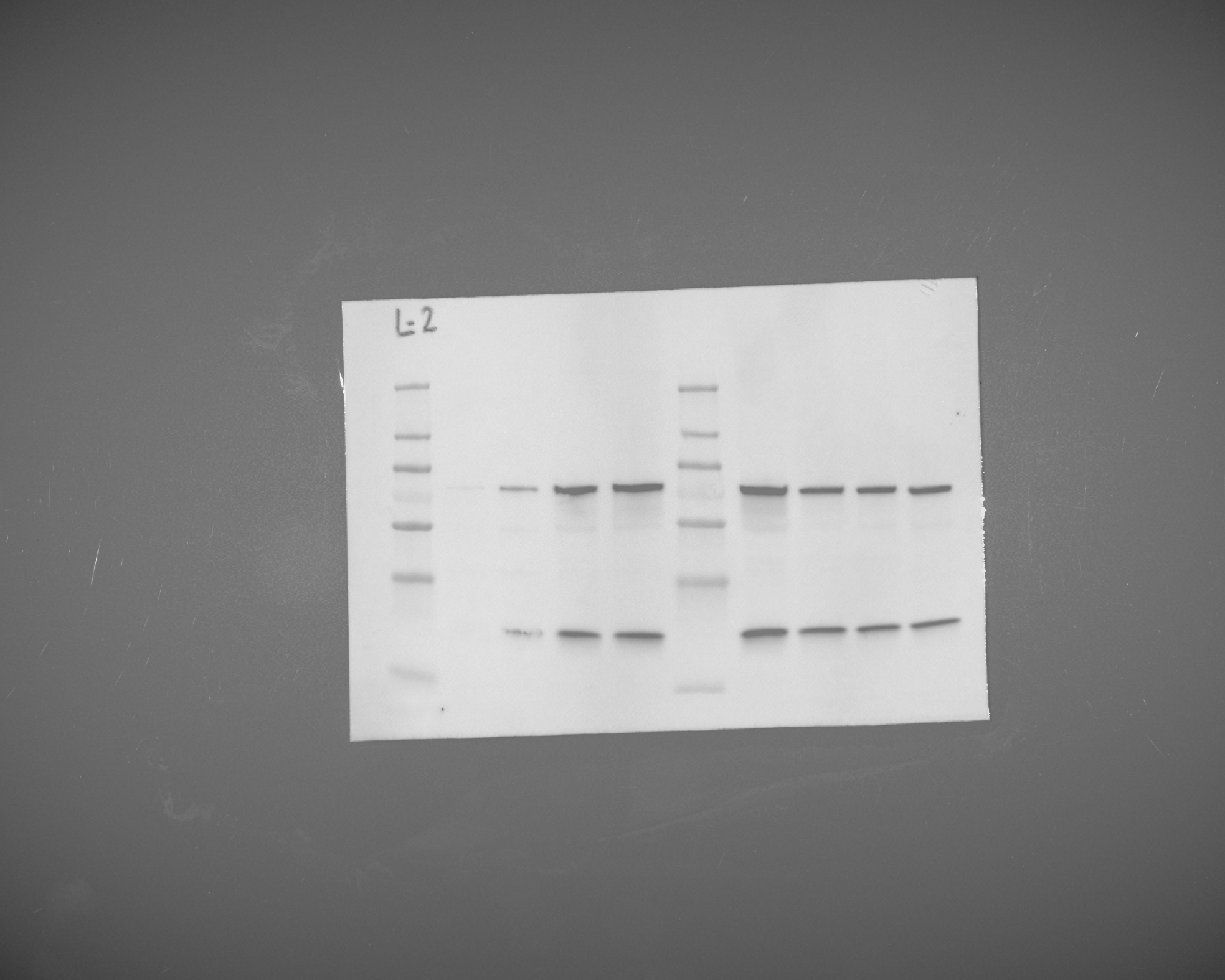

Supplement: Figure 3—source data 2. [file elife-100928-fig3-data2.zip › Figure 3 - Source data 2/3.2 D.Deretic 2024-04-04 17h02m10s(Composite).tif]

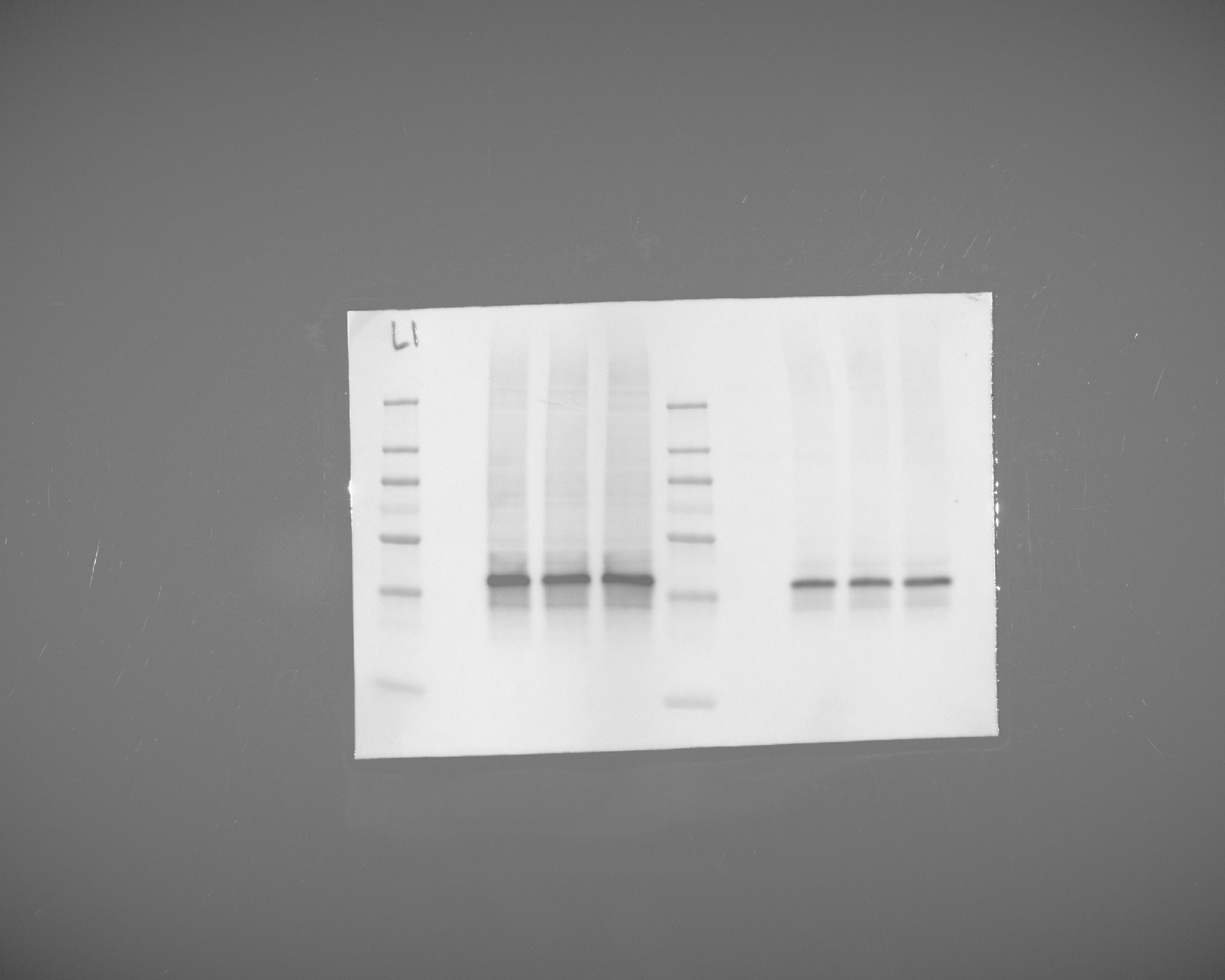

Supplement: Figure 3—source data 2. [file elife-100928-fig3-data2.zip › Figure 3 - Source data 2/3.3 D.Deretic 2024-04-04 16h54m46s(Composite).tif]

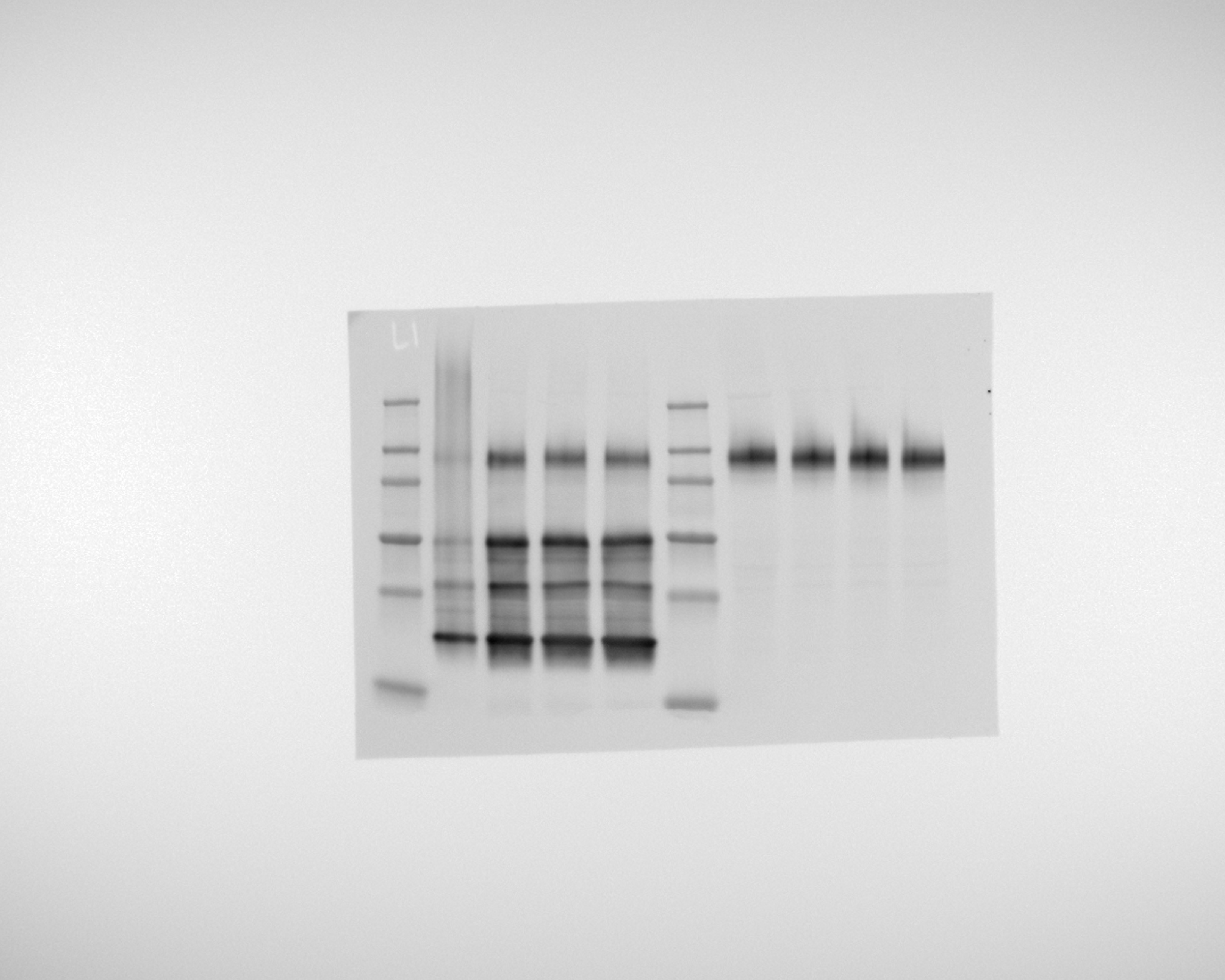

Supplement: Figure 3—source data 2. [file elife-100928-fig3-data2.zip › Figure 3 - Source data 2/3.4 D.Deretic 2024-04-04 16h55m09s(IRDye 680RD).tif]

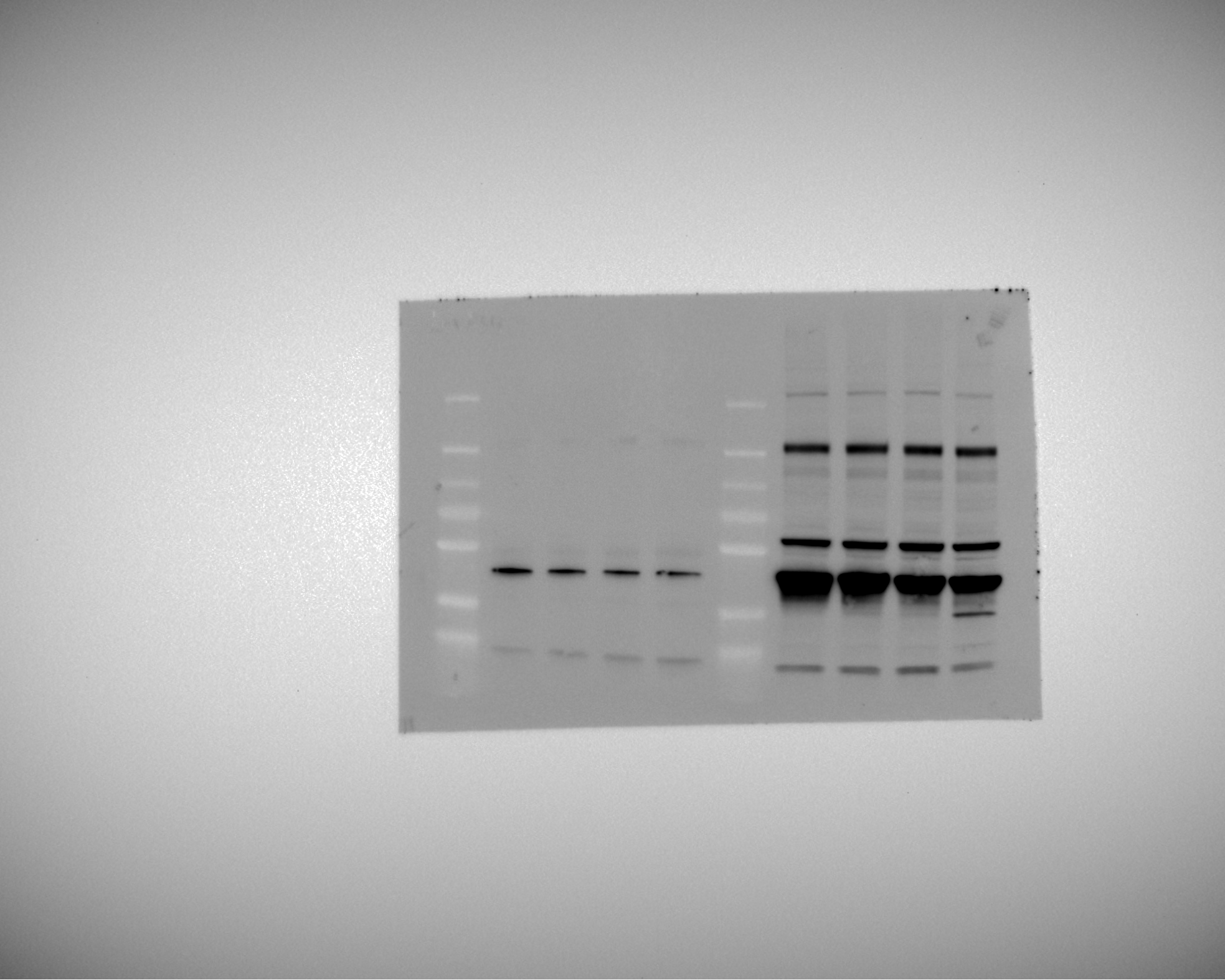

Supplement: Figure 3—source data 2. [file elife-100928-fig3-data2.zip › Figure 3 - Source data 2/3.5 D.Deretic 2024-04-04 16h55m13st(IRDye 800CW).tif]

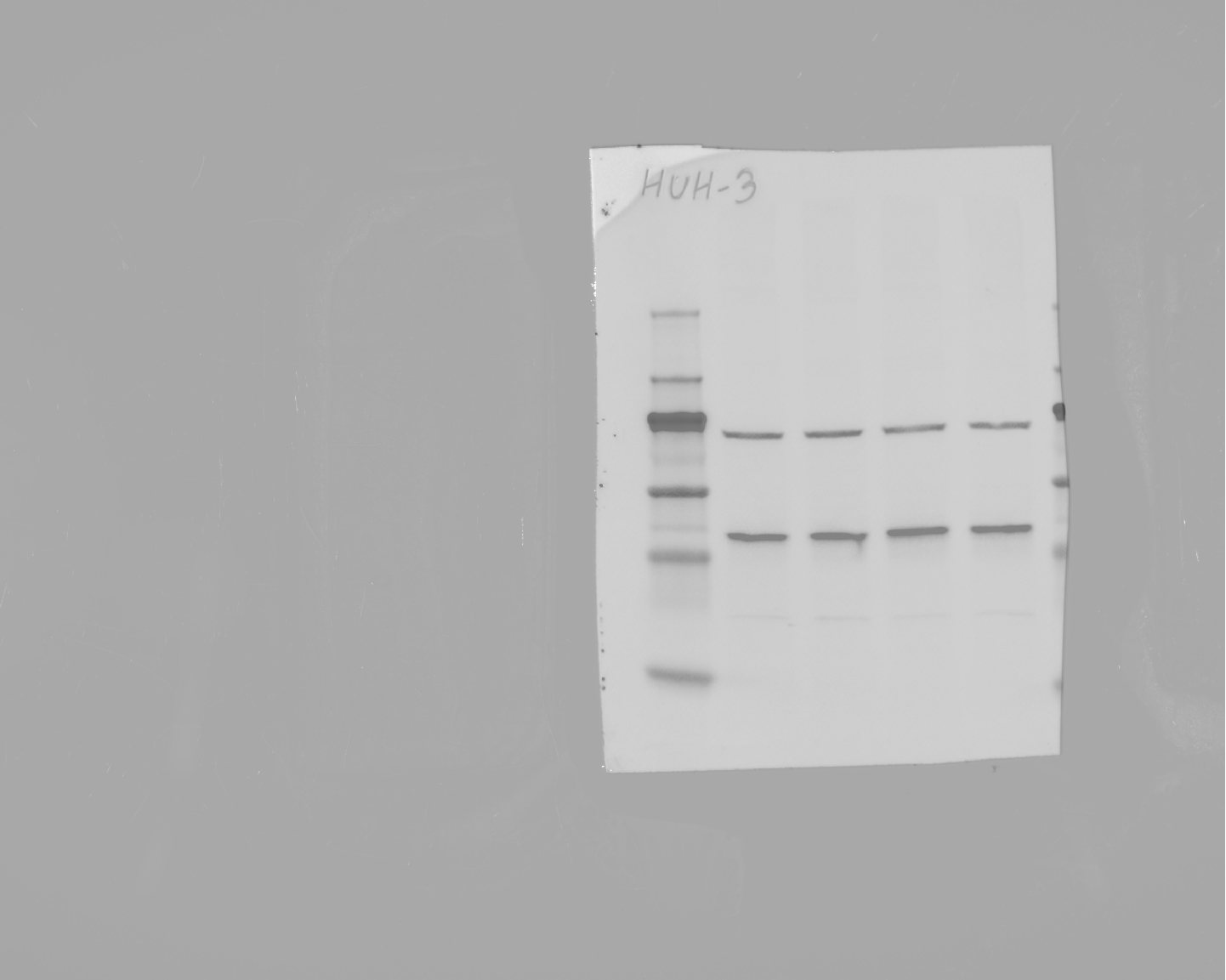

Supplement: Figure 4—figure supplement 1—source data 2. [file elife-100928-fig4-figsupp1-data2.zip › Figure 4-Figure Suppliment 1 - Source Data 2/1.1 2023-10-12 15h26m51s Deretic Lab(Composite).tif]

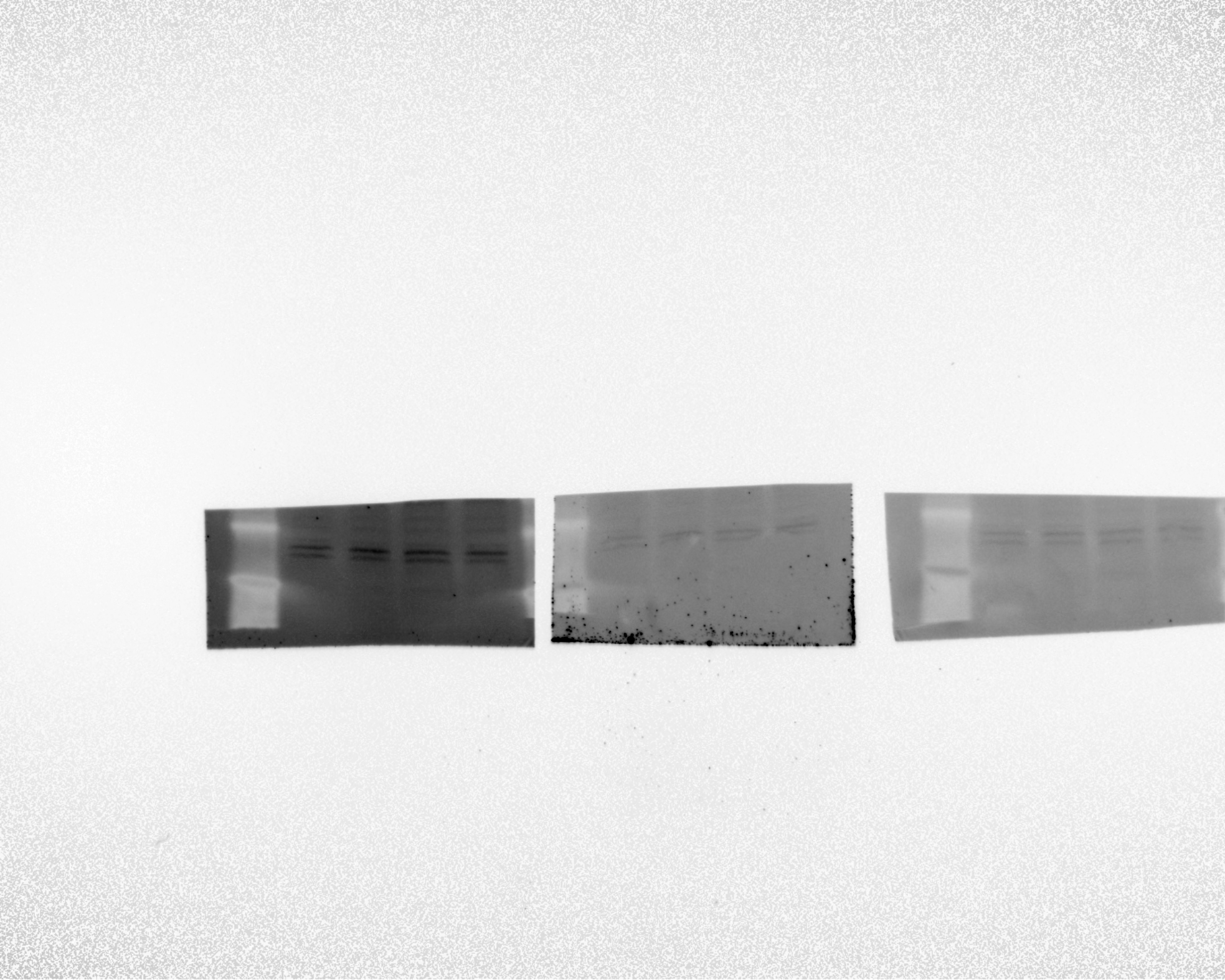

Supplement: Figure 4—figure supplement 1—source data 2. [file elife-100928-fig4-figsupp1-data2.zip › Figure 4-Figure Suppliment 1 - Source Data 2/1.2 D.Deretic 2023-11-15 17h20m09s(IRDye 680RD).tif]

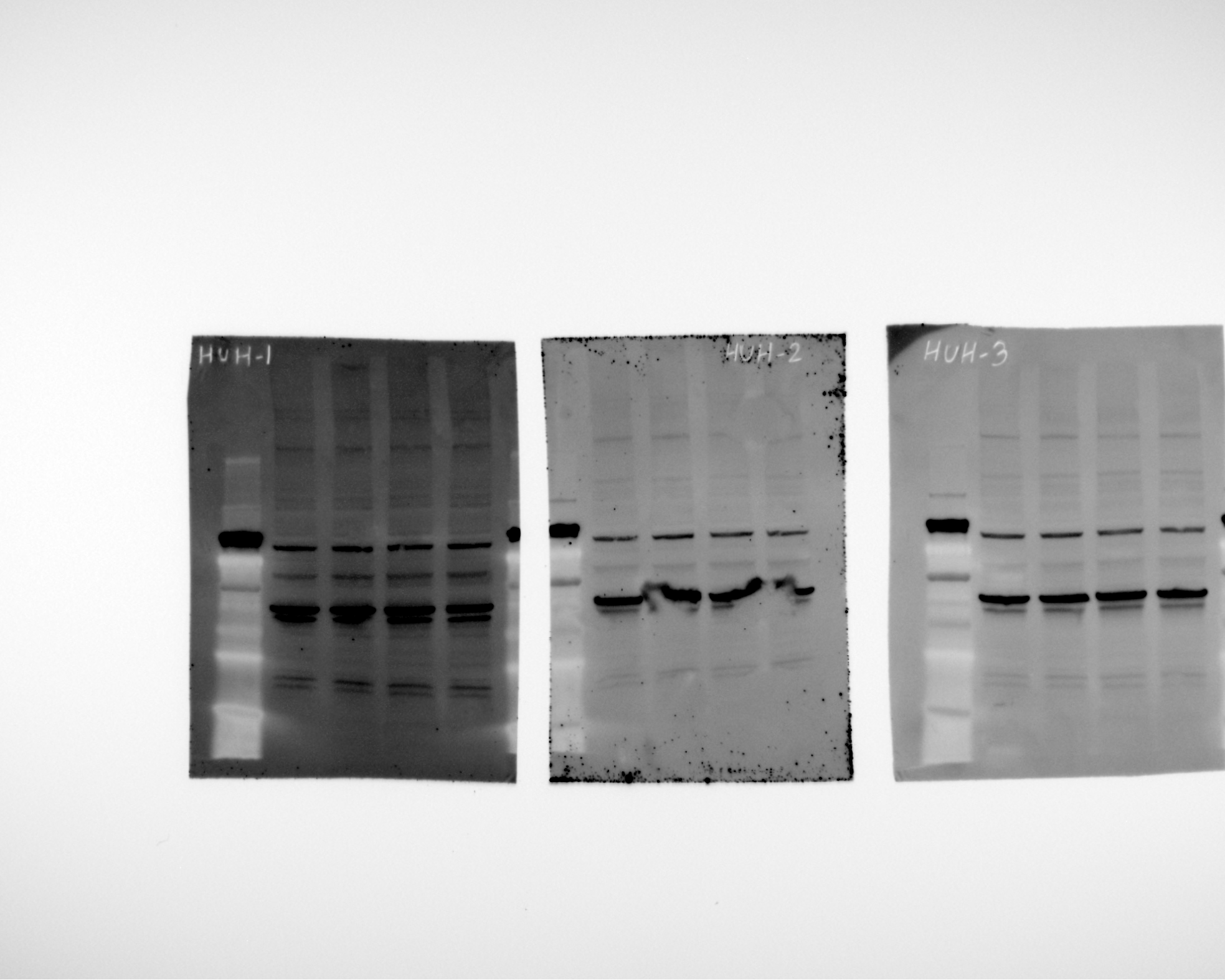

Supplement: Figure 4—figure supplement 1—source data 2. [file elife-100928-fig4-figsupp1-data2.zip › Figure 4-Figure Suppliment 1 - Source Data 2/1.3 D.Deretic 2023-11-15 17h17m50s(IRDye 680RD).tif]

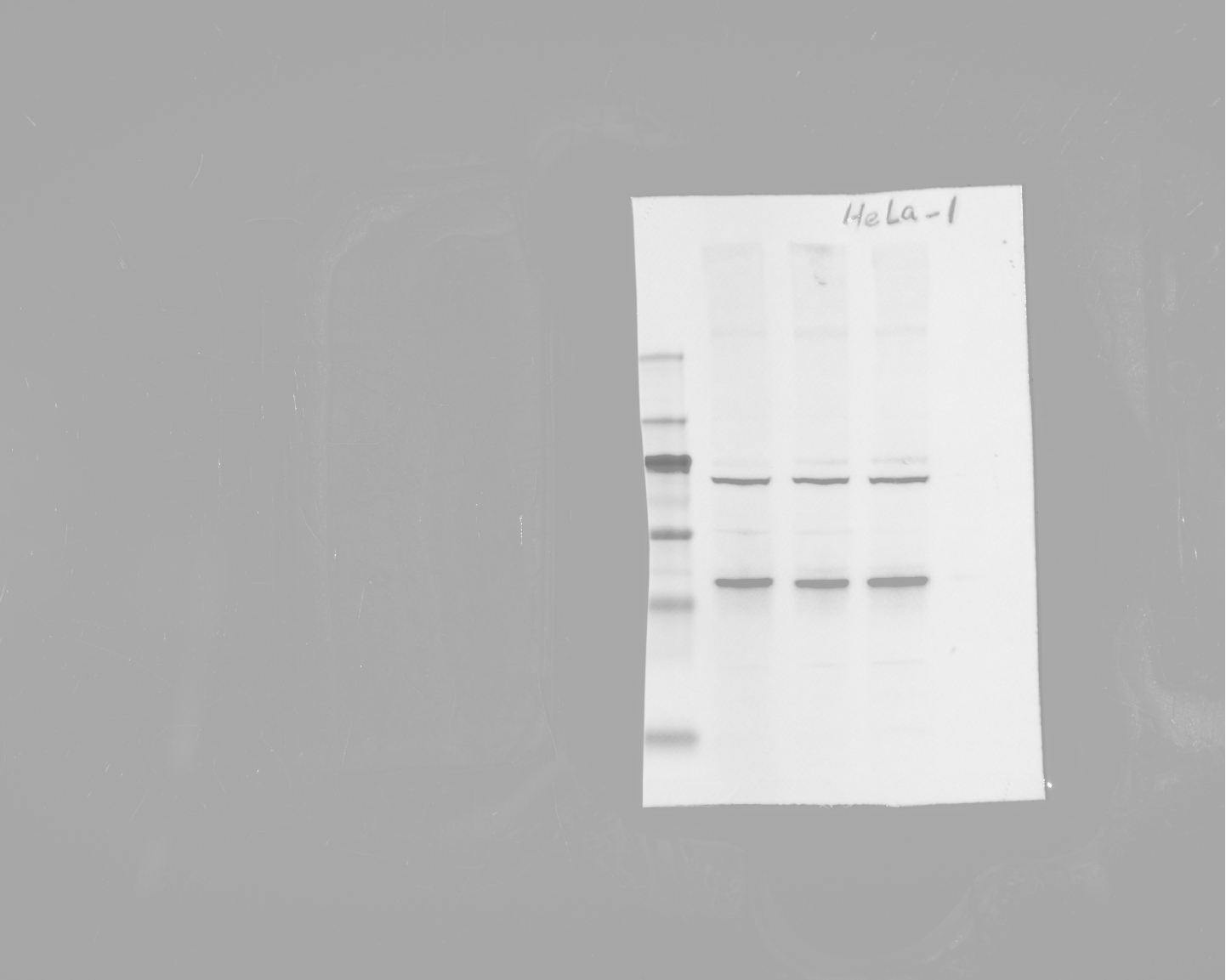

Supplement: Figure 4—figure supplement 1—source data 2. [file elife-100928-fig4-figsupp1-data2.zip › Figure 4-Figure Suppliment 1 - Source Data 2/2.1 2023-10-12 15h28m44s Deretic Lab(Composite).jpg]

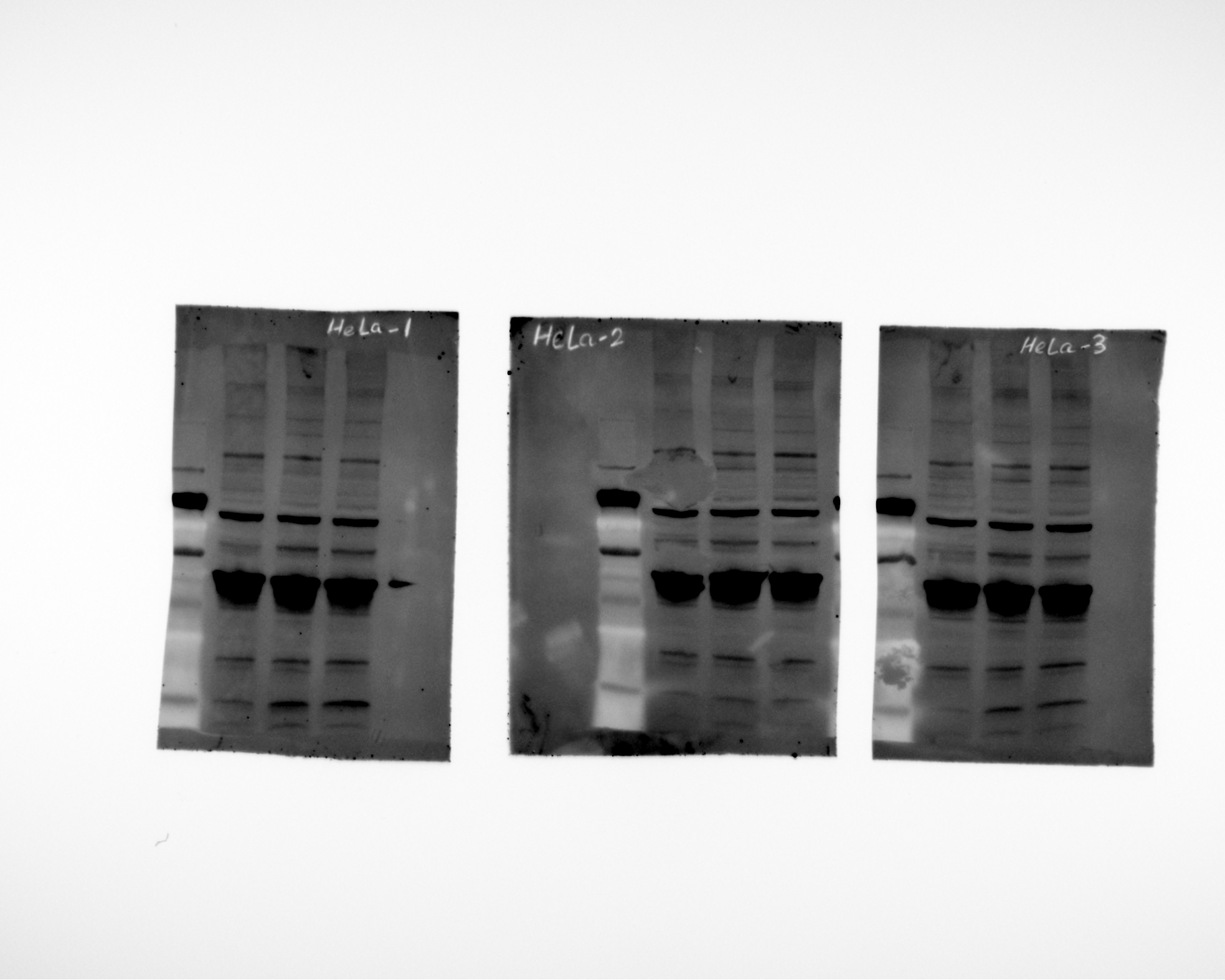

Supplement: Figure 4—figure supplement 1—source data 2. [file elife-100928-fig4-figsupp1-data2.zip › Figure 4-Figure Suppliment 1 - Source Data 2/2.2 D.Deretic 2023-11-15 17h08m53s(IRDye 680RD).tif]

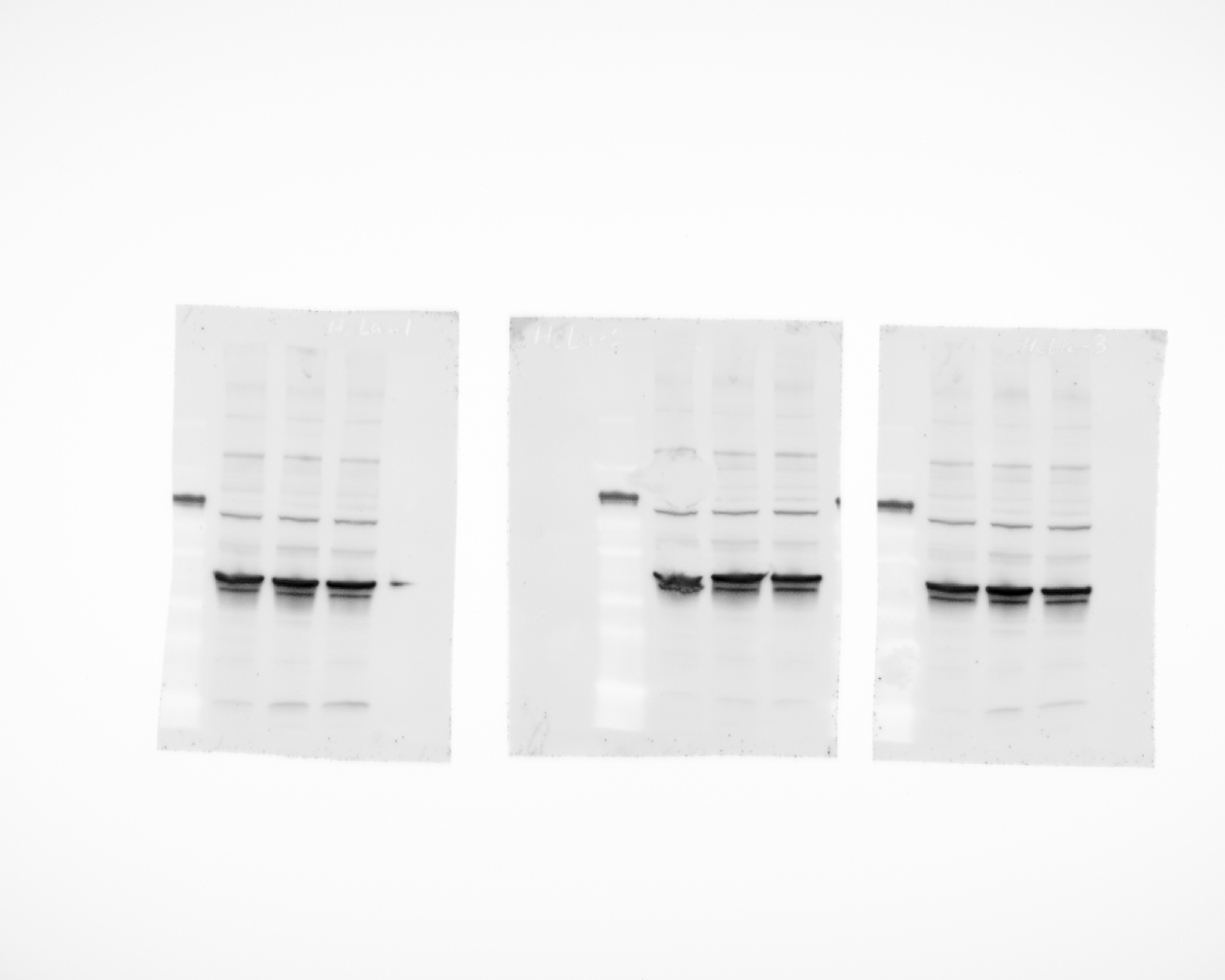

Supplement: Figure 4—figure supplement 1—source data 2. [file elife-100928-fig4-figsupp1-data2.zip › Figure 4-Figure Suppliment 1 - Source Data 2/2.3 D.Deretic 2023-11-15 17h10m34s(IRDye 800CW).tif]

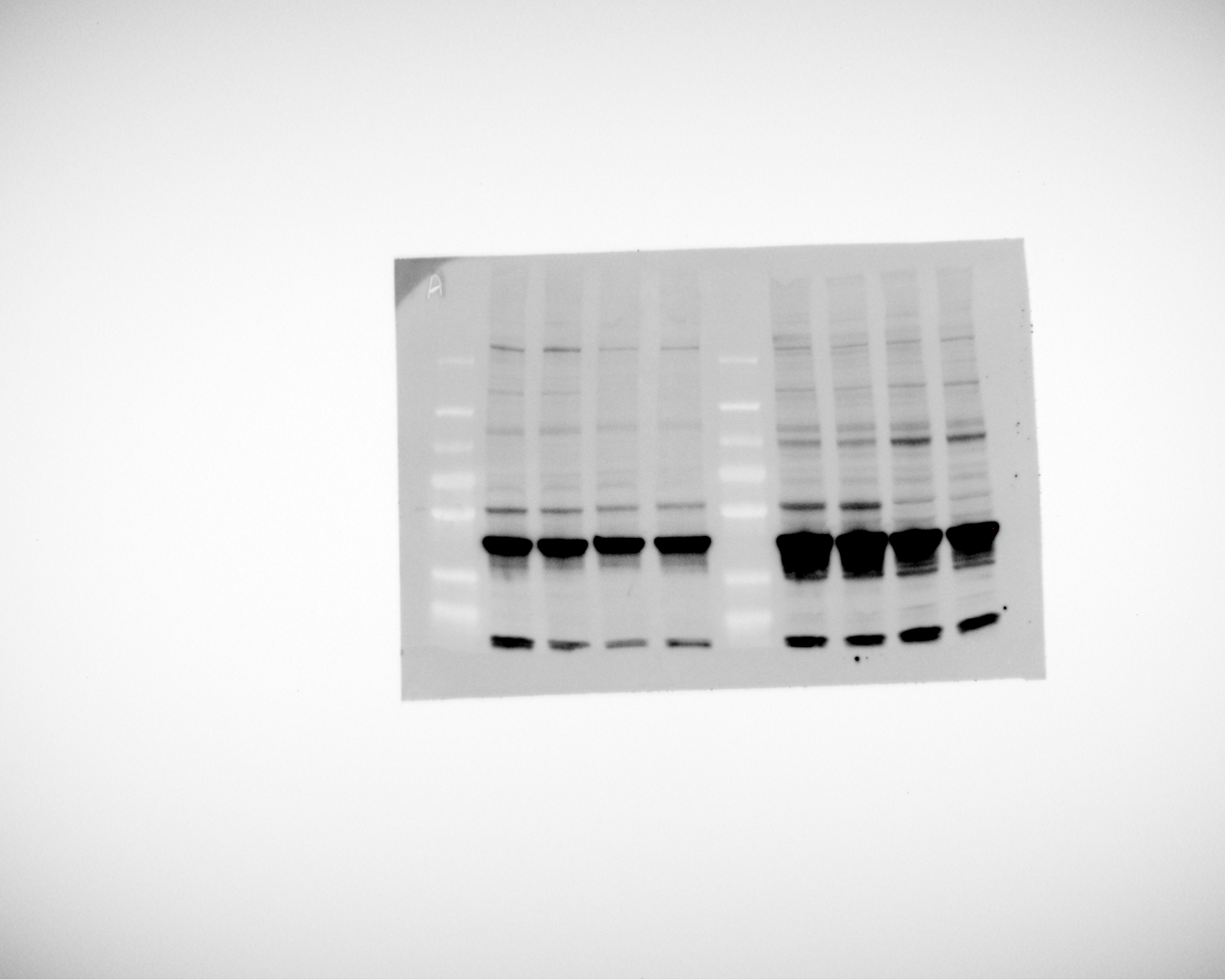

Supplement: Figure 4—figure supplement 1—source data 2. [file elife-100928-fig4-figsupp1-data2.zip › Figure 4-Figure Suppliment 1 - Source Data 2/3.1 D.Deretic 2024-04-30 16h54m39s(IRDye 800CW).tif]

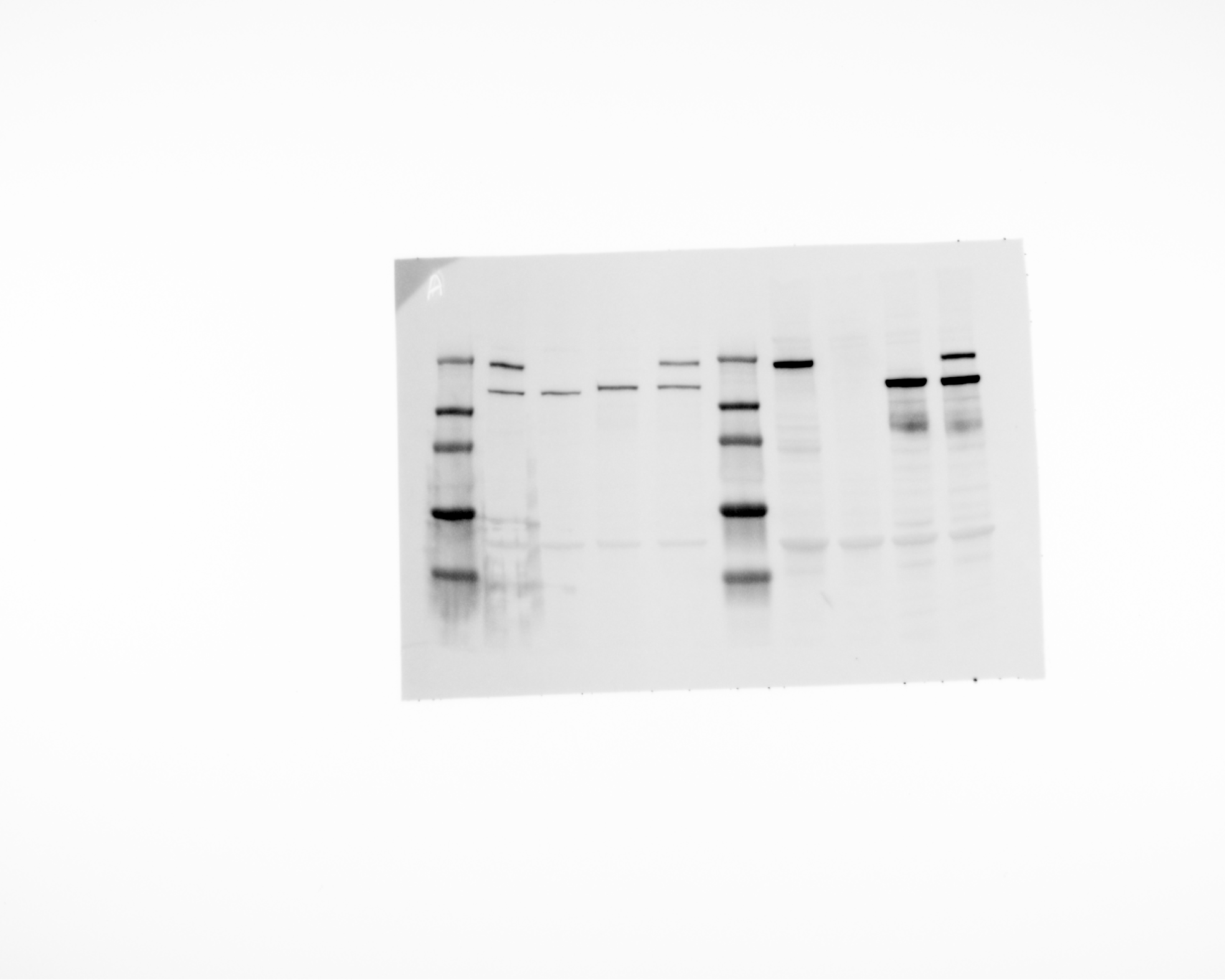

Supplement: Figure 4—figure supplement 1—source data 2. [file elife-100928-fig4-figsupp1-data2.zip › Figure 4-Figure Suppliment 1 - Source Data 2/3.2 D.Deretic 2024-04-30 16h53m50s(IRDye 680RD).tif]

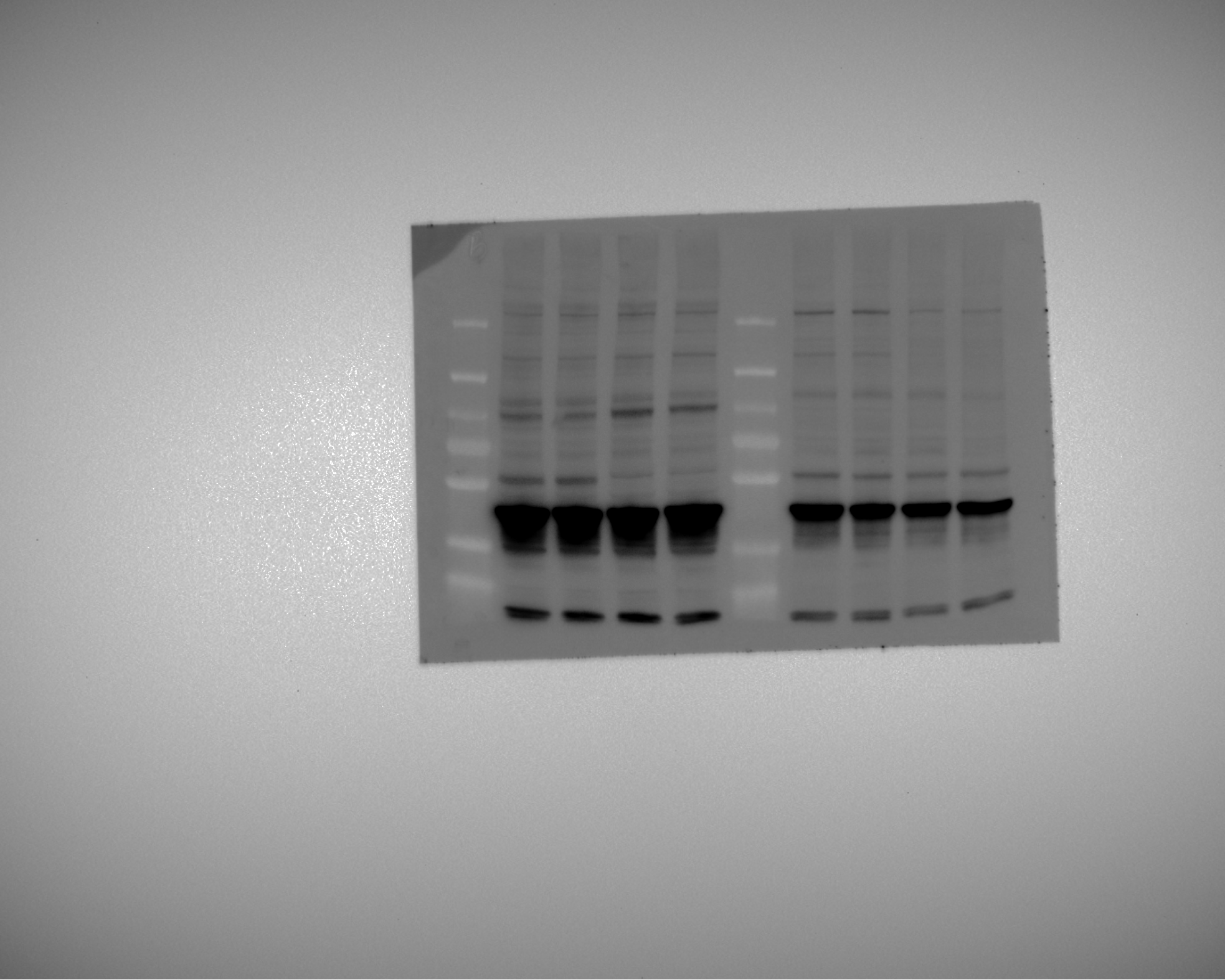

Supplement: Figure 4—figure supplement 1—source data 2. [file elife-100928-fig4-figsupp1-data2.zip › Figure 4-Figure Suppliment 1 - Source Data 2/3.3 D.Deretic 2024-04-30 16h57m03s(IRDye 800CW).tif]

1 to 4 : Lyso-IP Samples  
5 to 8 : Input Samples

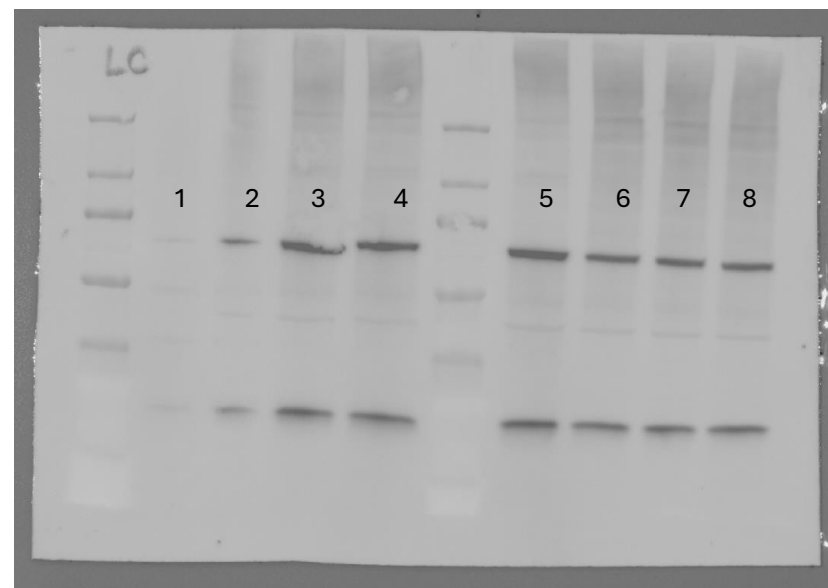

RAB7

Supplement: Figure 6—source data 1. [file elife-100928-fig6-data1.zip › Figure 6 - Source Data 1/Figure 6 - source data 1.1 uncropped and labelled.pdf]

1 to 4 : Lyso-IP Samples  
5 to 8 : Input Samples

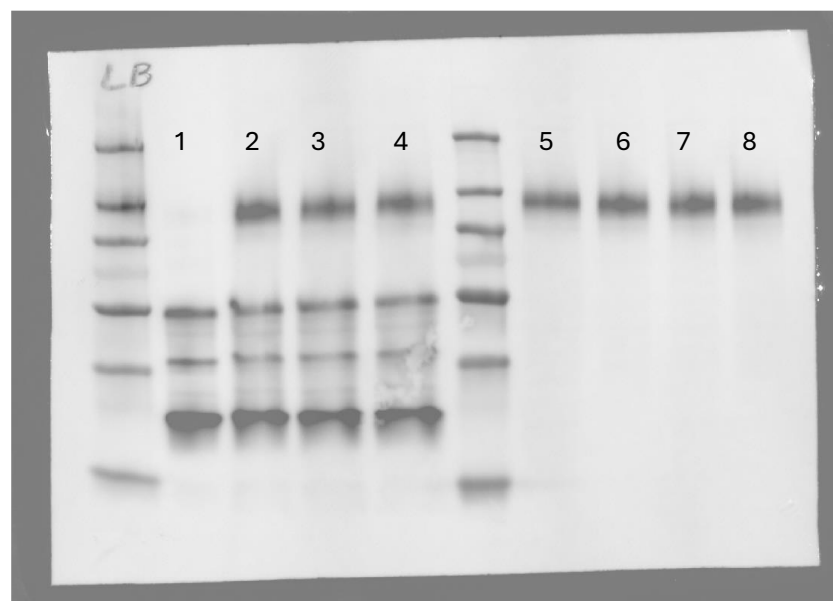

LAMP2

Supplement: Figure 6—source data 1. [file elife-100928-fig6-data1.zip › Figure 6 - Source Data 1/Figure 6 - source data 1.2 uncropped and labelled.pdf]

1 to 4 : Lyso-IP Samples  
5 to 8 : Input Samples

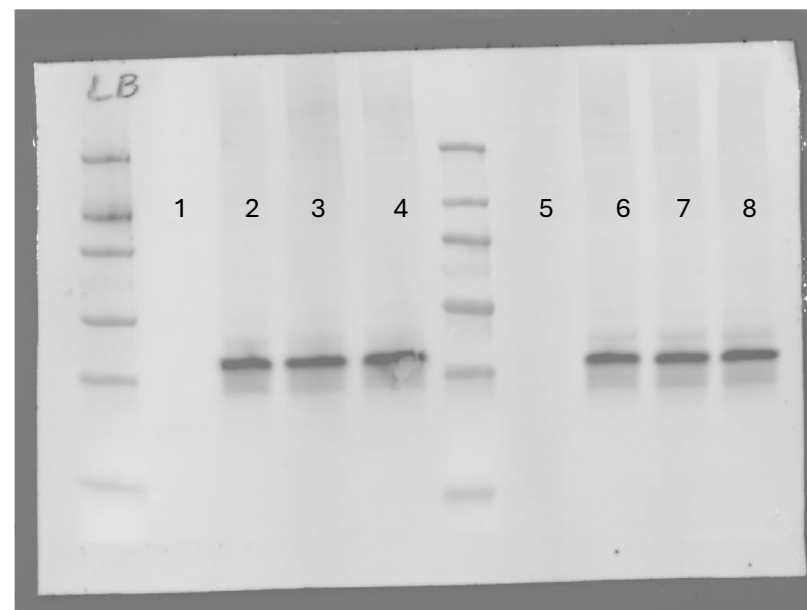

TMEM192-3xHA

Supplement: Figure 6—source data 1. [file elife-100928-fig6-data1.zip › Figure 6 - Source Data 1/Figure 6 - source data 1.3 uncropped and labelled.pdf]

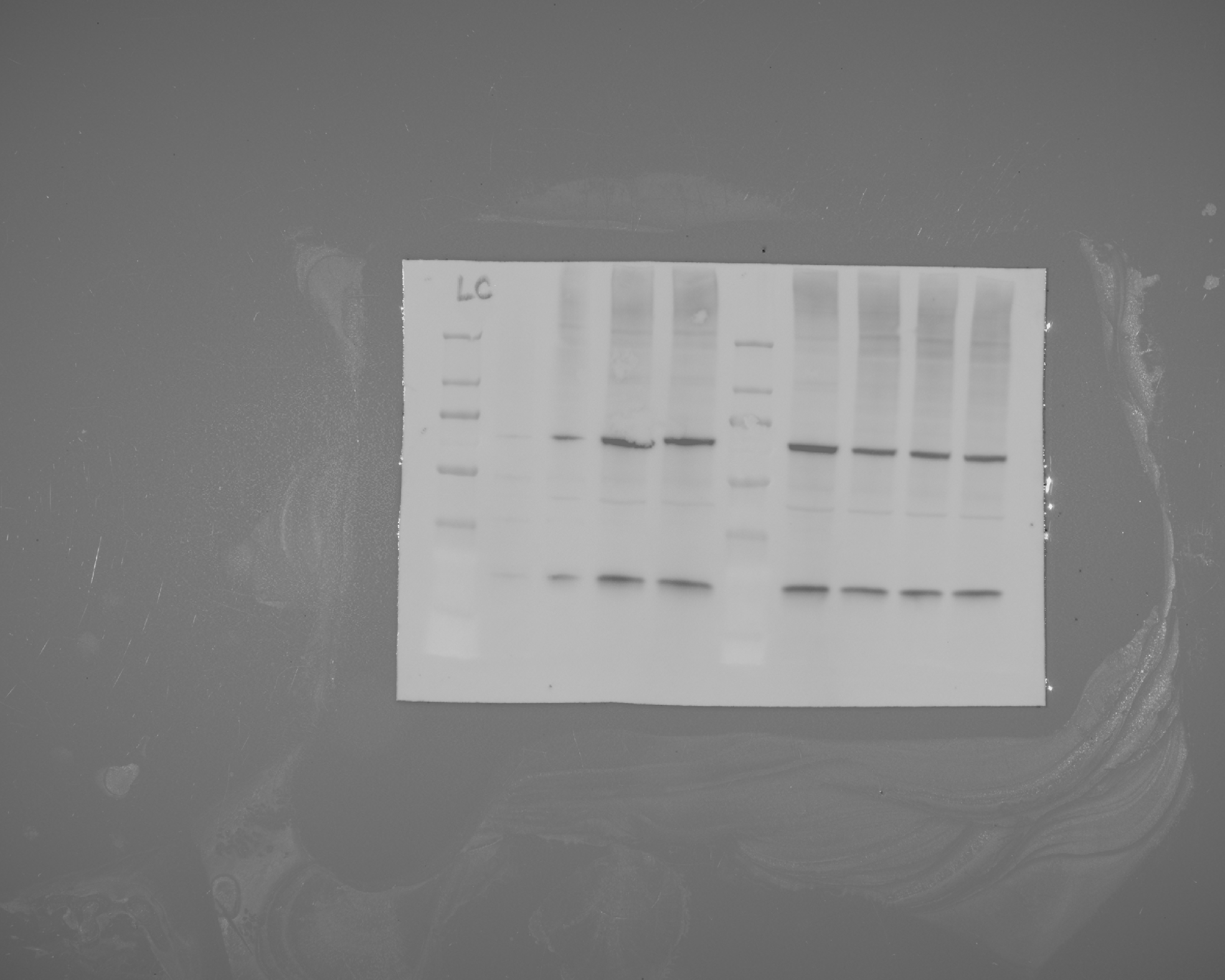

Supplement: Figure 6—source data 2. [file elife-100928-fig6-data2.zip › Figure 6 - Source Data 2/1.1 D.Deretic 2024-04-16 18h30m47s(Composite).tif]

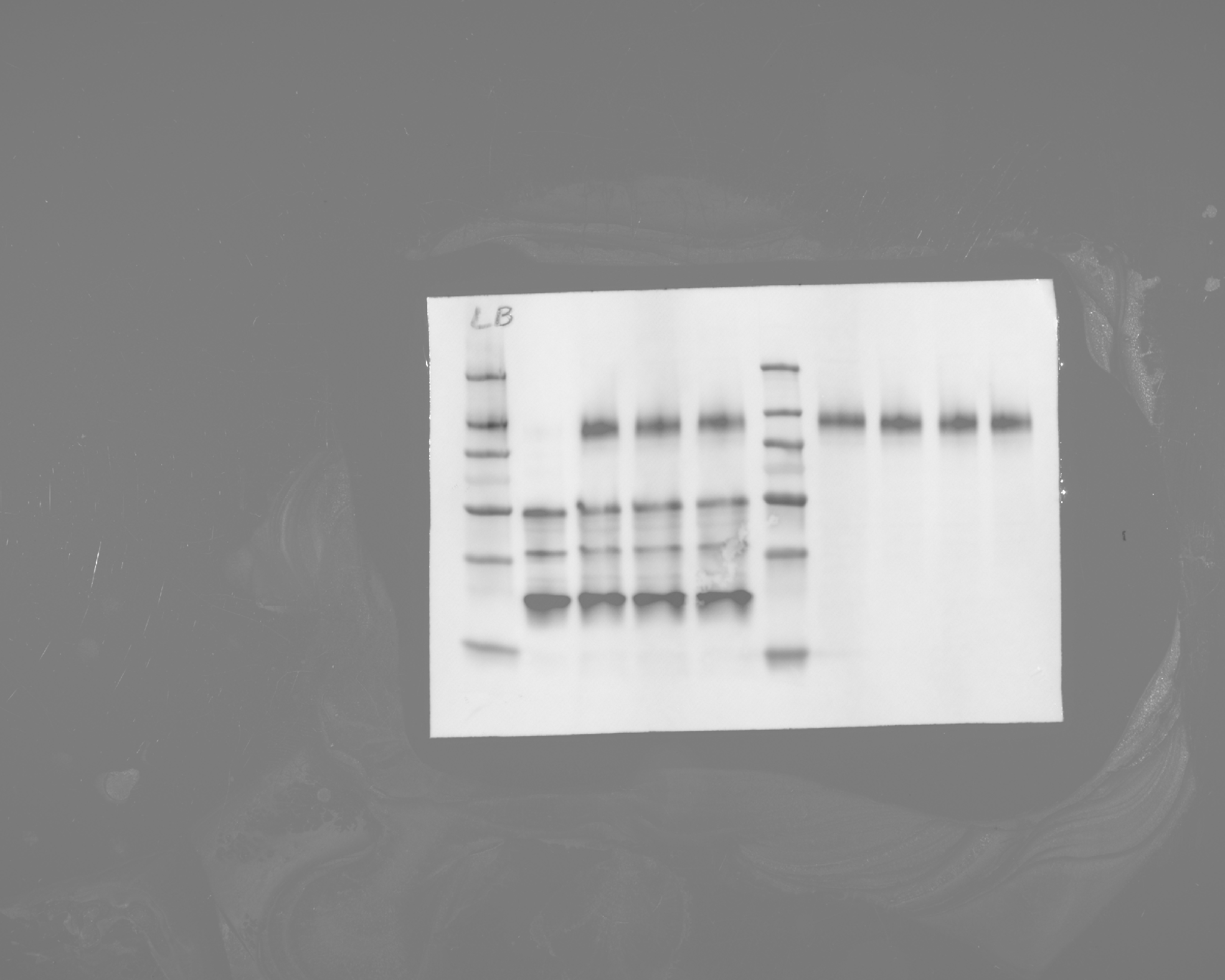

Supplement: Figure 6—source data 2. [file elife-100928-fig6-data2.zip › Figure 6 - Source Data 2/1.2 D.Deretic 2024-04-16 18h24m49s(Composite).tif]

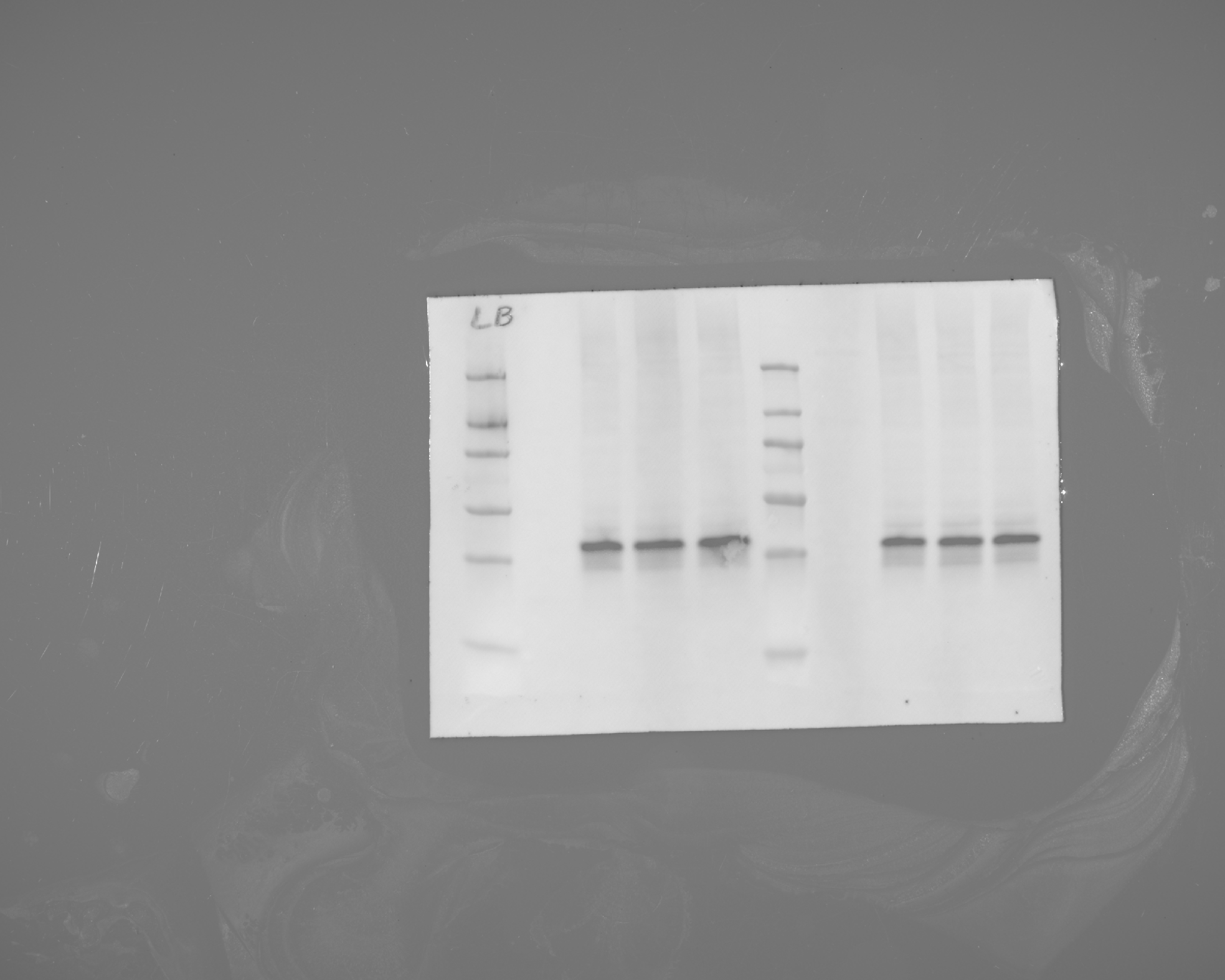

Supplement: Figure 6—source data 2. [file elife-100928-fig6-data2.zip › Figure 6 - Source Data 2/1.3 D.Deretic 2024-04-16 18h25m03s(Composite).tif]

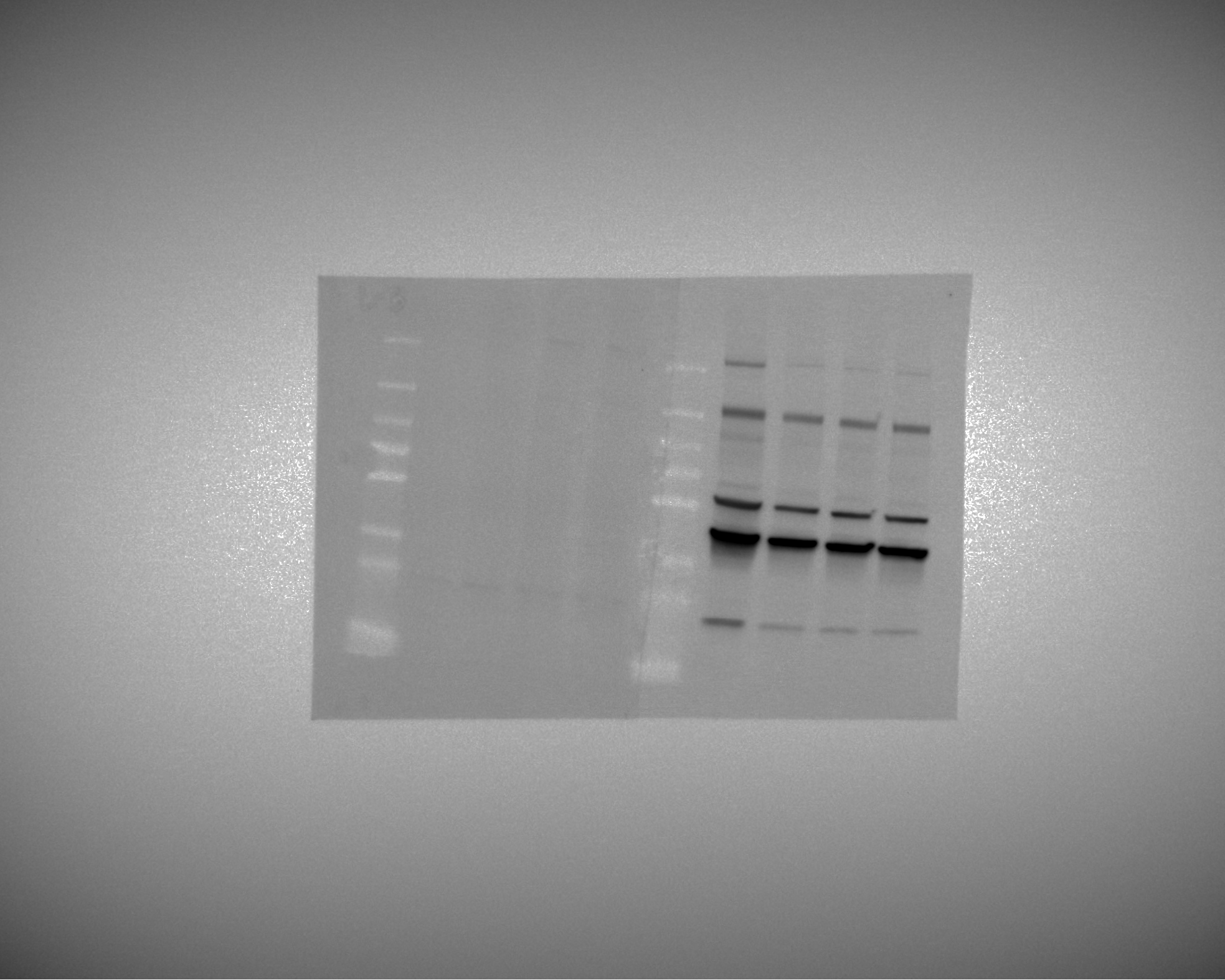

Supplement: Figure 6—source data 2. [file elife-100928-fig6-data2.zip › Figure 6 - Source Data 2/1.4 D.Deretic 2024-04-16 17h10m00s(IRDye 800CW).tif]

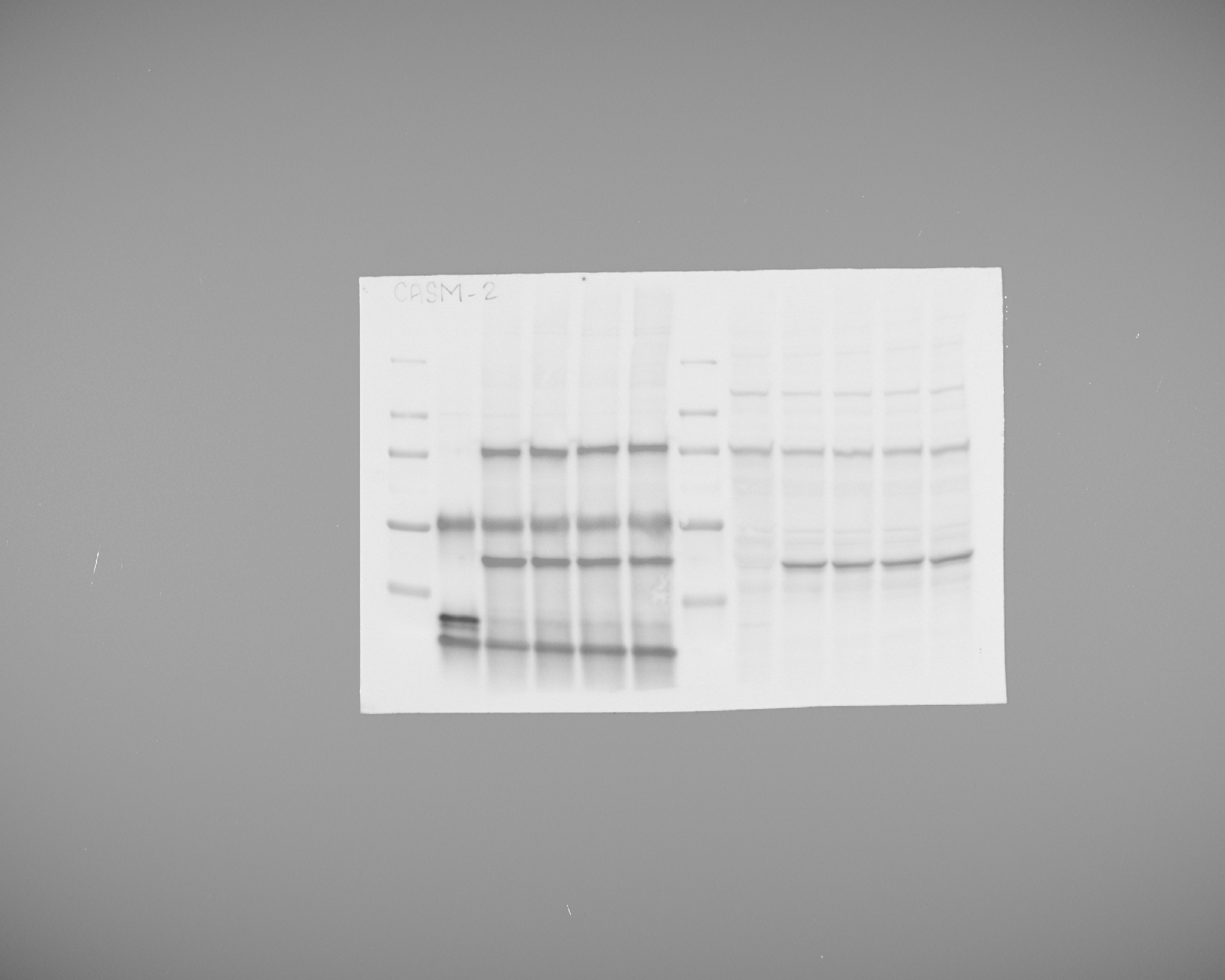

Supplement: Figure 7—figure supplement 1—source data 2. [file elife-100928-fig7-figsupp1-data2.zip › Figure 7 - Figure Suppliment 1 - Source data 2/1.1 D.Deretic 2024-05-22 17h34m47s(Composite).tif]

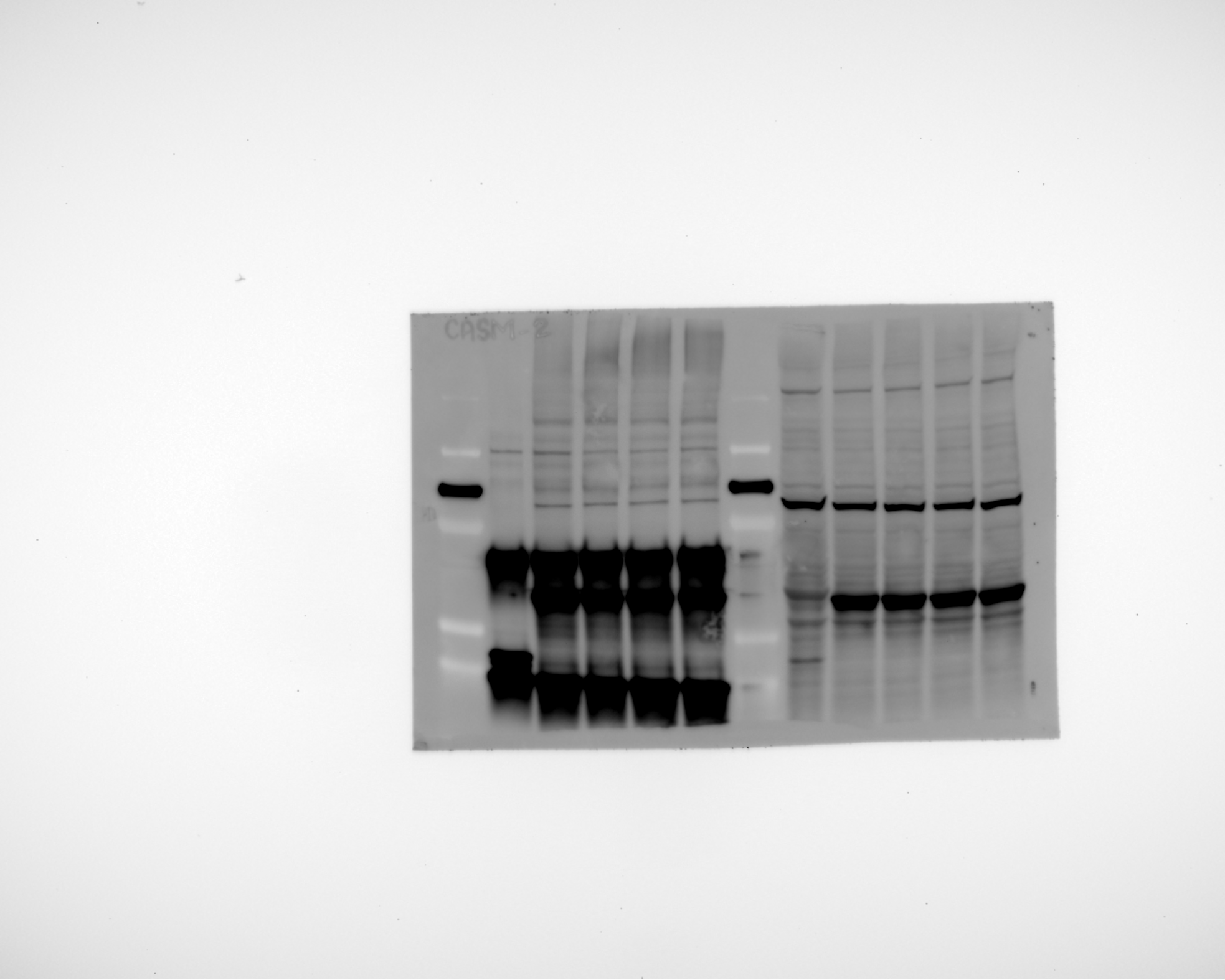

Supplement: Figure 7—figure supplement 1—source data 2. [file elife-100928-fig7-figsupp1-data2.zip › Figure 7 - Figure Suppliment 1 - Source data 2/1.2 D.Deretic 2024-05-23 17h43m17s(IRDye 800CW).tif]

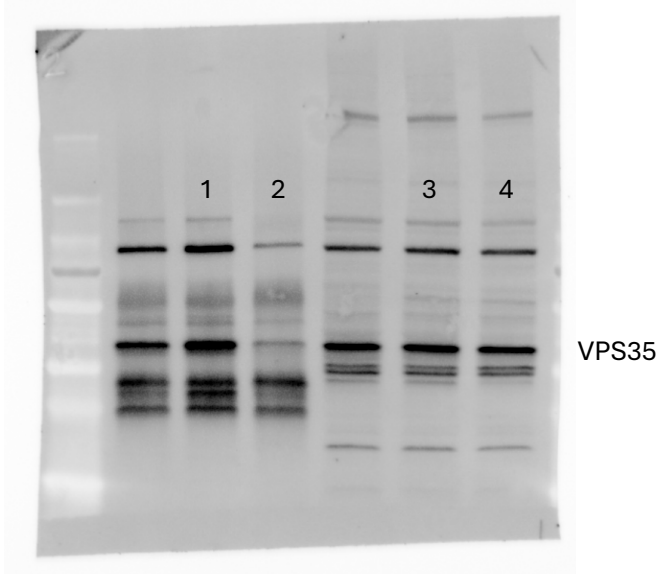

1 and 2 : CO-IP Samples  
3 and 4 : Input Samples

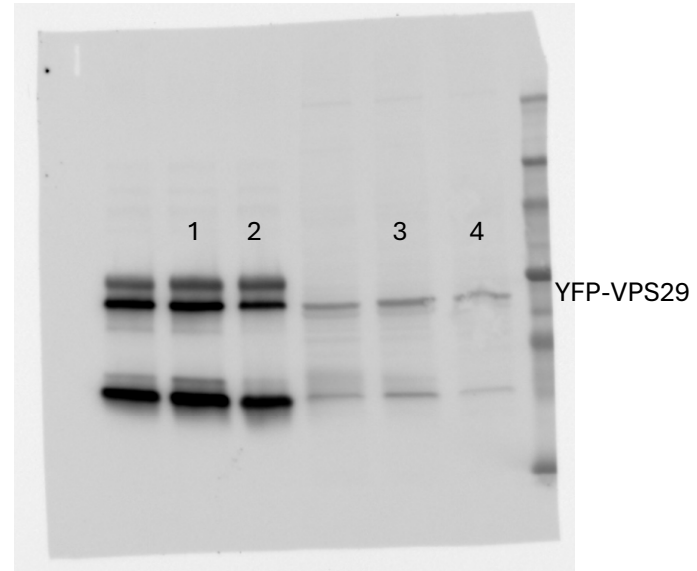

1 and 2 : CO-IP Samples  
3 and 4 : Input Samples

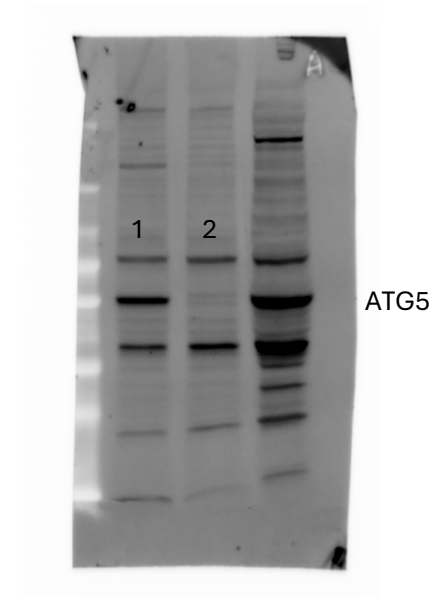

1 : HeLa-WT  
2 : HeLa ATG5-KO

Supplement: Figure 8—figure supplement 3—source data 1. [file elife-100928-fig8-figsupp3-data1.zip › Figure 8 - Figure Suppliment 3 - Source data 1/Figure Supplimentary 10 - source data 2.1 uncropped and labelled.pdf]

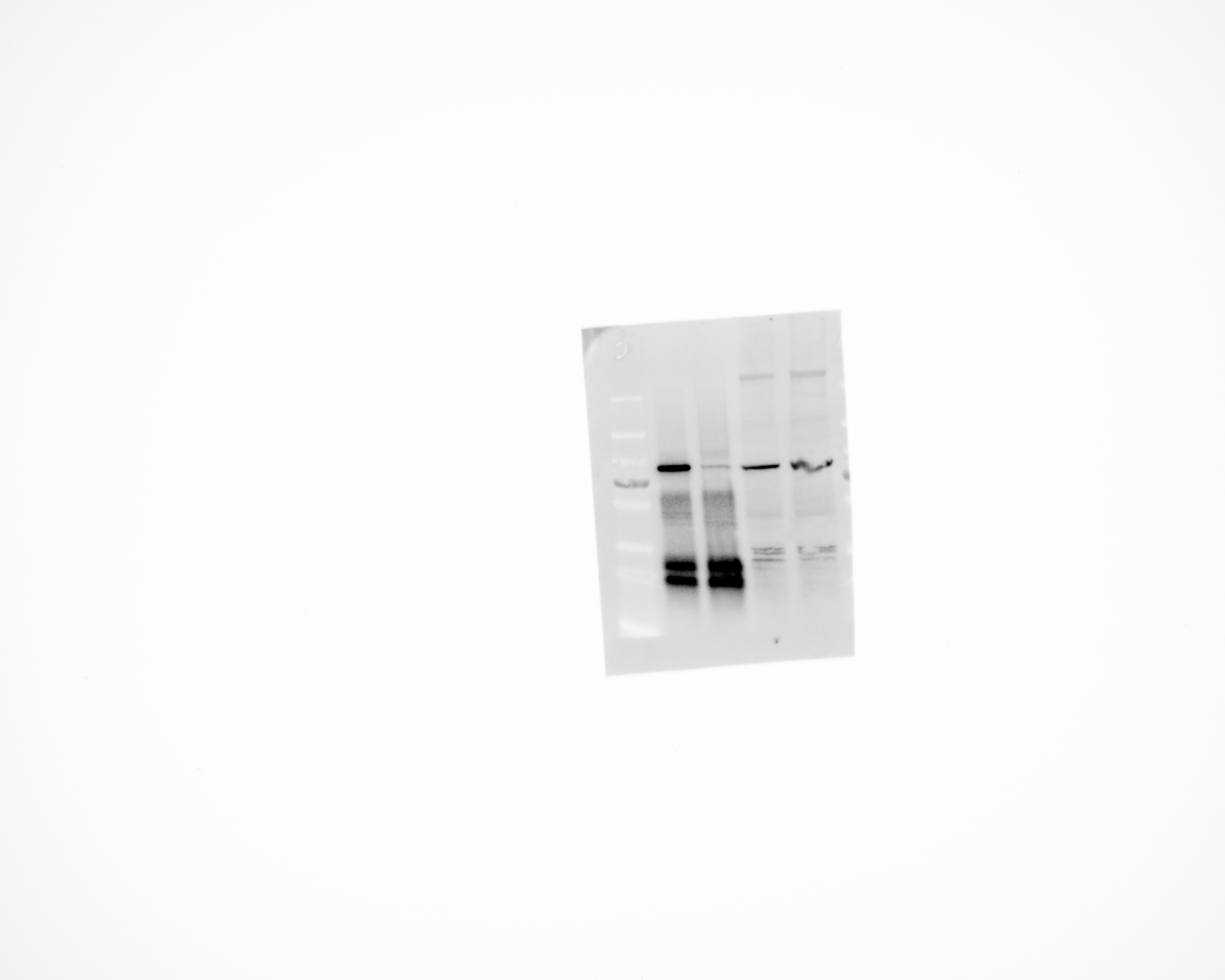

Supplement: Figure 8—figure supplement 3—source data 2. [file elife-100928-fig8-figsupp3-data2.zip › Figure 8 - Figure Suppliment 3 - Source data 2/1.1 2023-07-06 16h47m52s Deretic Lab(IRDye 800CW).tif]

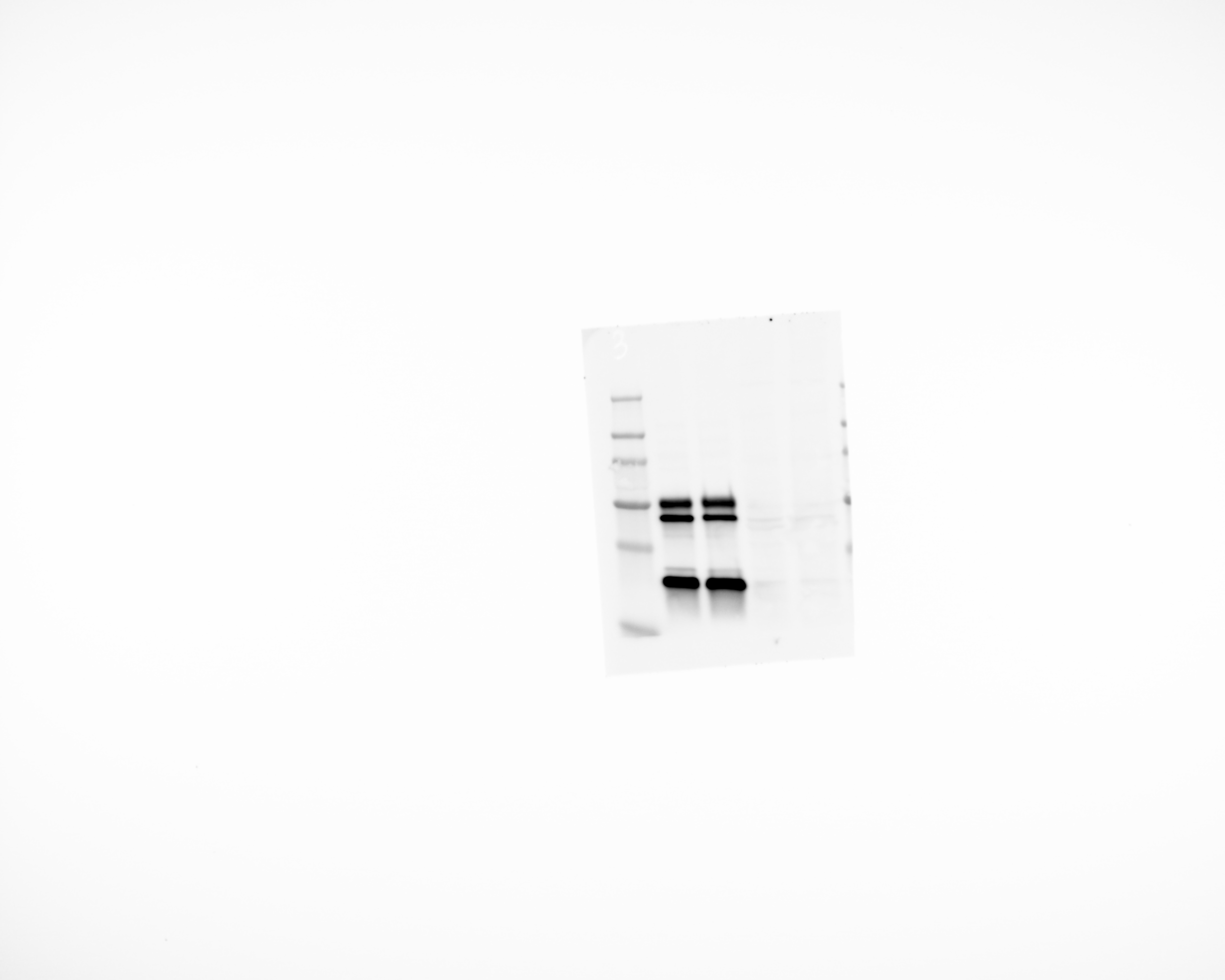

Supplement: Figure 8—figure supplement 3—source data 2. [file elife-100928-fig8-figsupp3-data2.zip › Figure 8 - Figure Suppliment 3 - Source data 2/1.2 2023-07-06 16h48m34s Deretic Lab(IRDye 680RD).tif]

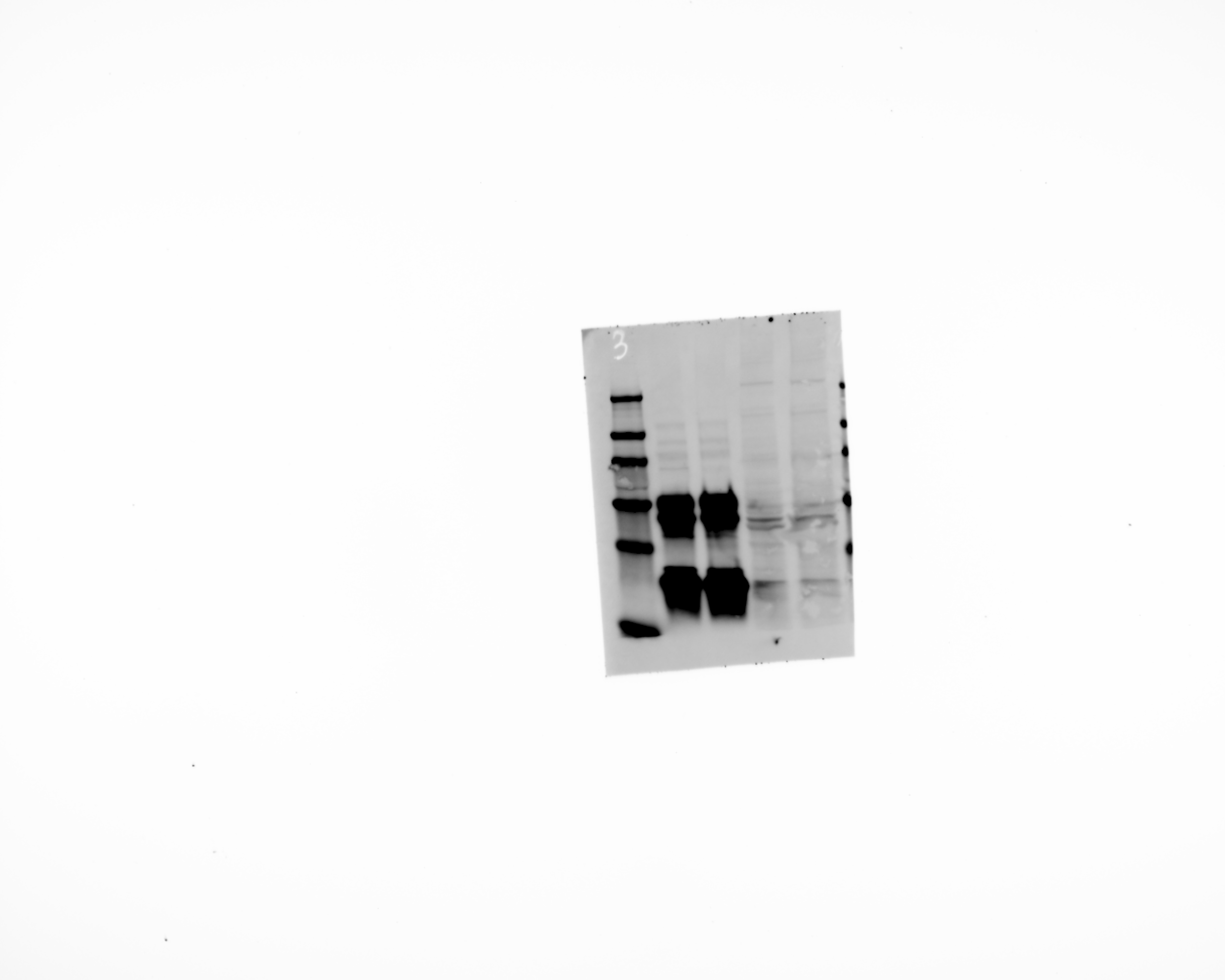

Supplement: Figure 8—figure supplement 3—source data 2. [file elife-100928-fig8-figsupp3-data2.zip › Figure 8 - Figure Suppliment 3 - Source data 2/1.2.1 2023-07-06 16h49m24s Deretic Lab(IRDye 680RD).tif]

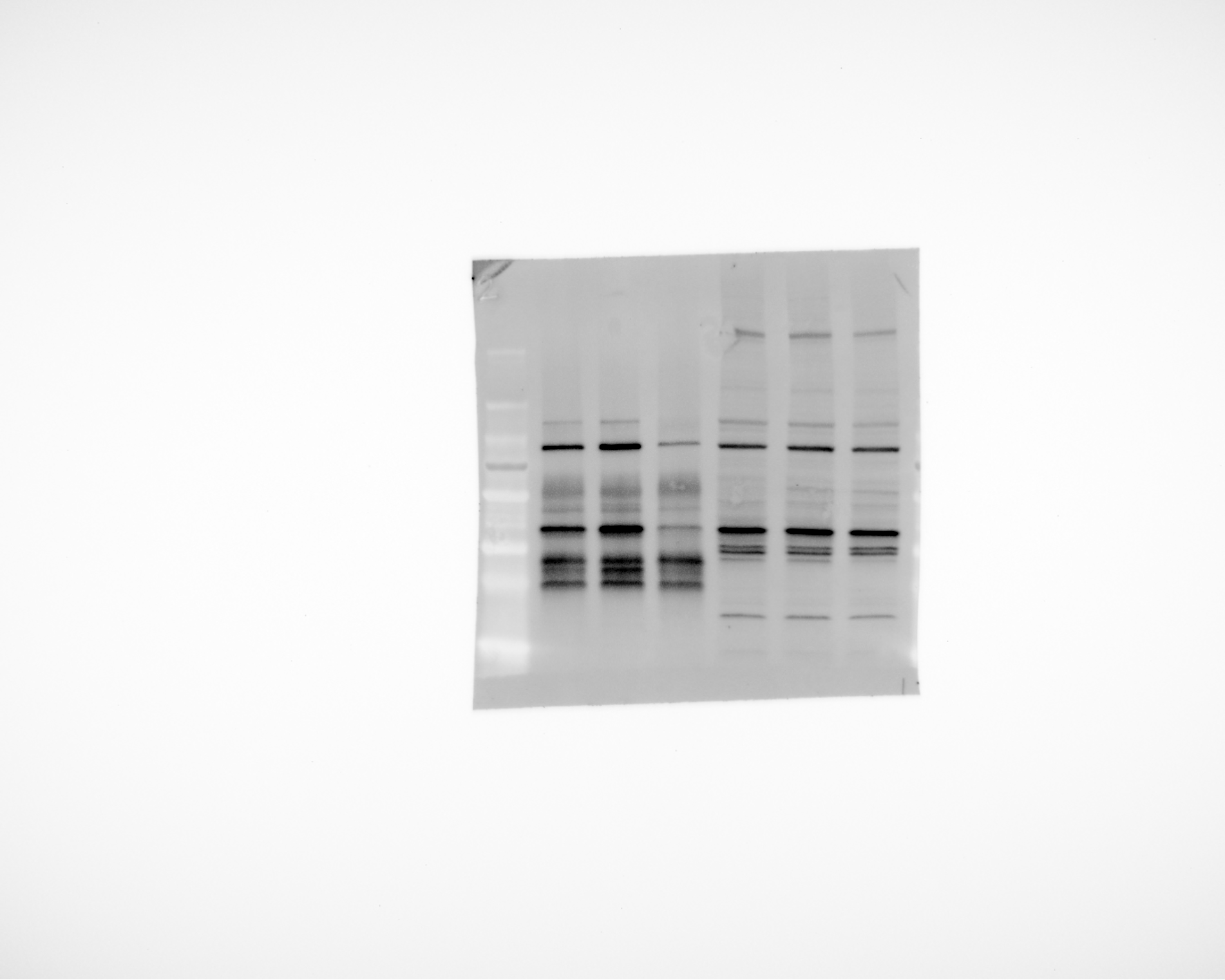

Supplement: Figure 8—figure supplement 3—source data 2. [file elife-100928-fig8-figsupp3-data2.zip › Figure 8 - Figure Suppliment 3 - Source data 2/2.1 2023-11-08 17h50m13s Deretic Lab(IRDye 800CW).tif]

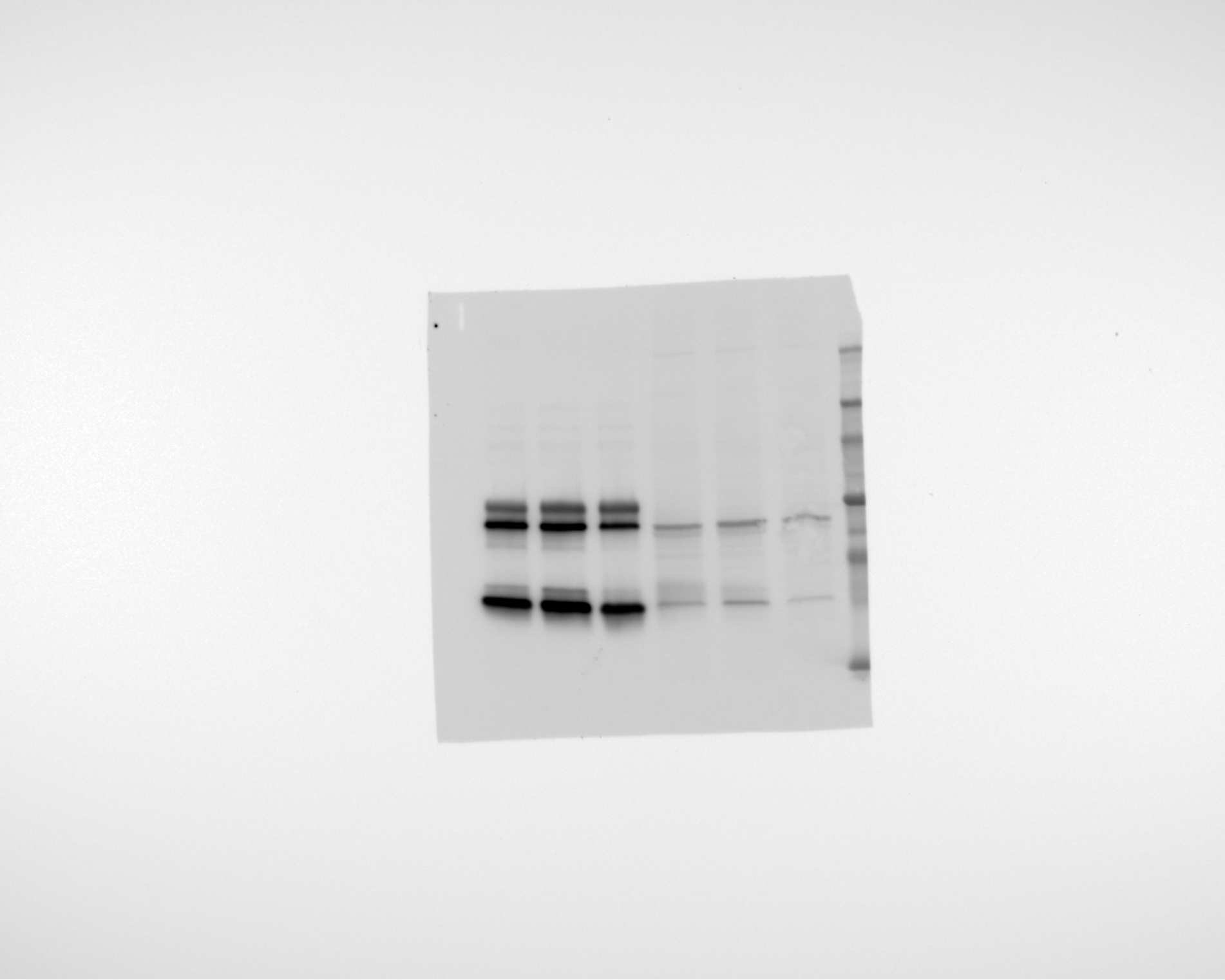

Supplement: Figure 8—figure supplement 3—source data 2. [file elife-100928-fig8-figsupp3-data2.zip › Figure 8 - Figure Suppliment 3 - Source data 2/2.2 2023-11-07 14h47m41s Deretic Lab(IRDye 680RD).tif]

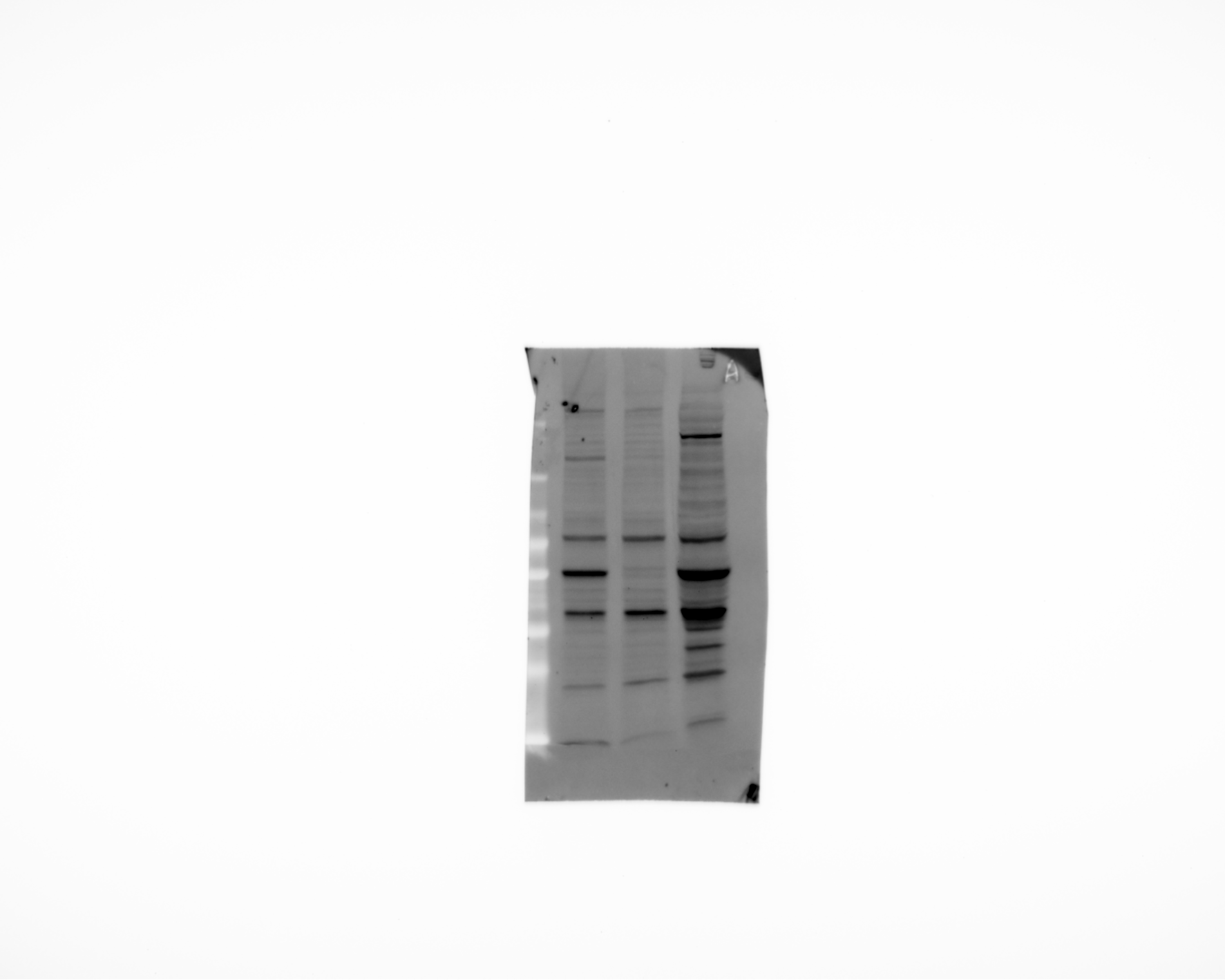

Supplement: Figure 8—figure supplement 3—source data 2. [file elife-100928-fig8-figsupp3-data2.zip › Figure 8 - Figure Suppliment 3 - Source data 2/2.3 2023-11-07 14h44m19s Deretic Lab(IRDye 800CW).tif]
