# Supplementary material for: Noncanonical roles of ATG5 and membrane atg8ylation in retromer assembly and function
Source: eLife. 2025 Jan 7;13:RP100928. doi: 10.7554/eLife.100928 (PMC11706607; doi:10.7554/eLife.100928)
Supplement: Figure 4—figure supplement 1—source data 1. [file elife-100928-fig4-figsupp1-data1.zip › Figure 4 - Figure Suppliment 1 - Source Data 1/Figure 4 - Fig Supplimentary 1 - source data 1.2.pdf]

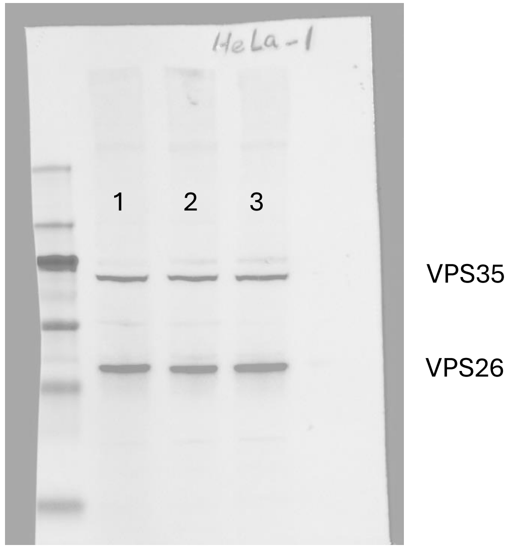

Lane Details:
Lane 1 : Huh7 WT
Lane 2 : Huh7 ATG5^{KO}
Lane 3 : Huh7 ATG5^{KO}

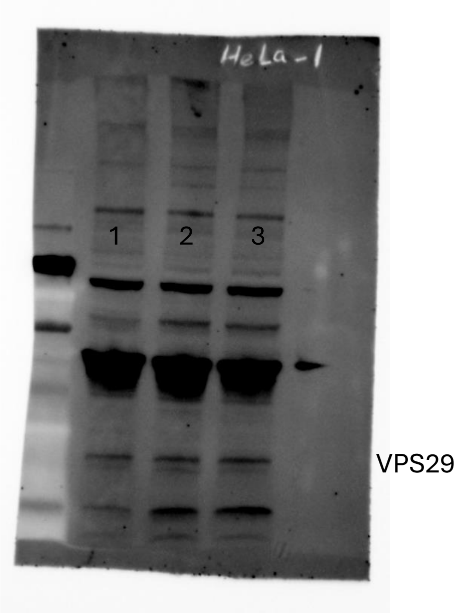

Lane Details:
Lane 1 : Huh7 WT
Lane 2 : Huh7 ATG5^{KO}
Lane 3 : Huh7 ATG5^{KO}

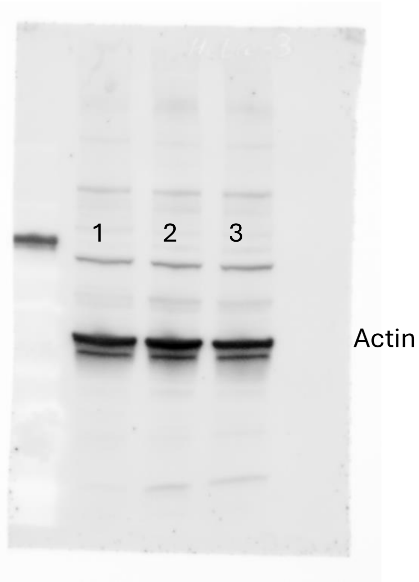

Lane Details:
Lane 1 : Huh7 WT
Lane 2 : Huh7 ATG5^{KO}
Lane 3 : Huh7 ATG5^{KO}
