# Supplementary material for: Noncanonical roles of ATG5 and membrane atg8ylation in retromer assembly and function
Source: eLife. 2025 Jan 7;13:RP100928. doi: 10.7554/eLife.100928 (PMC11706607; doi:10.7554/eLife.100928)
Supplement: Figure 6—source data 1. [file elife-100928-fig6-data1.zip › Figure 6 - Source Data 1/Figure 6 - source data 1.4 uncropped and labelled.pdf]

1 to 4 : Lyso-IP Samples
5 to 8 : Input Samples

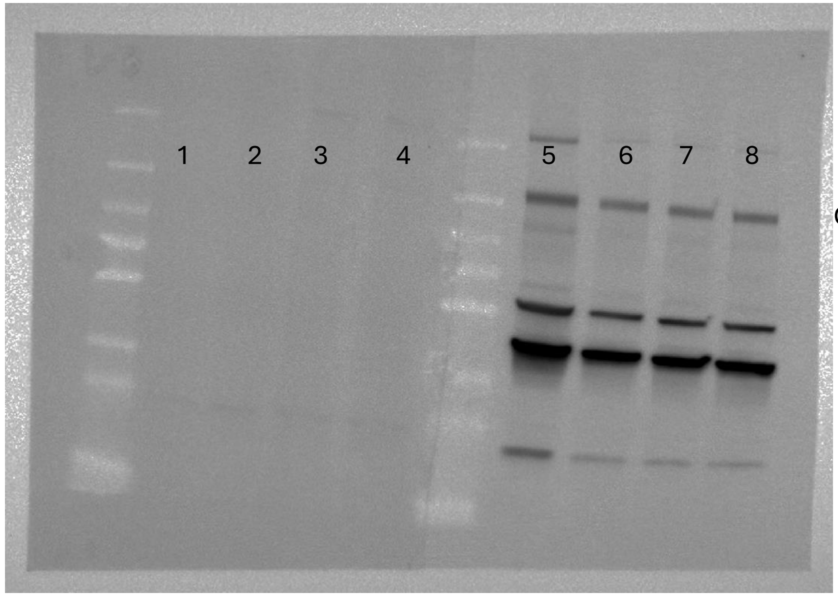

GM130 (Golgi)

PDI (ER)

TOMM20 (Mitochondria)
