# Supplementary material for: Noncanonical roles of ATG5 and membrane atg8ylation in retromer assembly and function
Source: eLife. 2025 Jan 7;13:RP100928. doi: 10.7554/eLife.100928 (PMC11706607; doi:10.7554/eLife.100928)
Supplement: Figure 7—figure supplement 1—source data 1. [file elife-100928-fig7-figsupp1-data1.zip › Figure 7 - Figure Suppliment 1 - Source data 1/Figure 7 - Suppliment 1 - source data 1.2.pdf]

1 to 5 : CO-IP Samples
6 to 10 : Input Samples

Transfection details:
Lane 1 : pDEST eGFP
Lane 2-5 : pDEST eGFP-LC3A

Treatment details:
Lane 1 and 2 : No treatment
Lane 3 : Bafilomycin(100nM)
Lane 4 : LLOMe (100uM)
Lane 5 : Monensin (100uM)

Treatment duration:
The treatment was given for 45 minutes

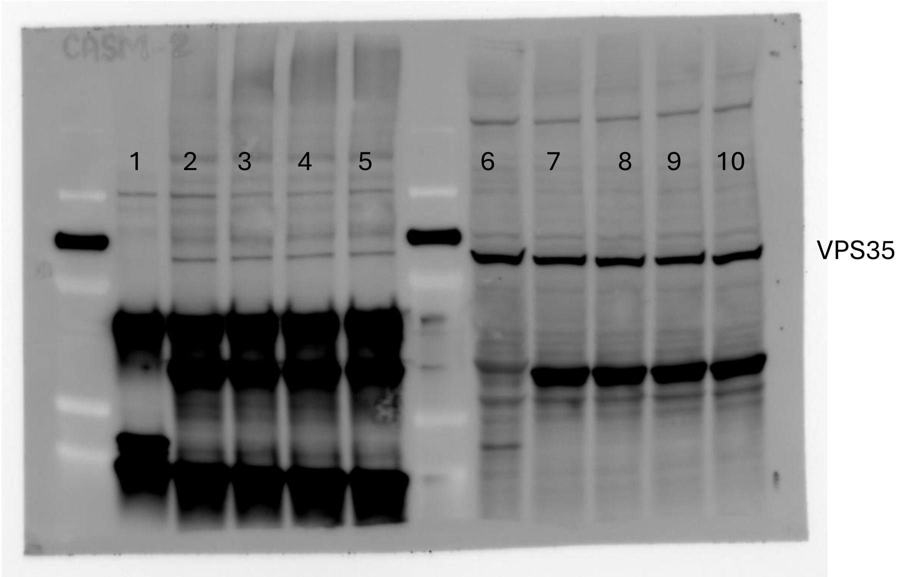

VPS35
