# Supplementary material for: Noncanonical roles of ATG5 and membrane atg8ylation in retromer assembly and function
Source: eLife. 2025 Jan 7;13:RP100928. doi: 10.7554/eLife.100928 (PMC11706607; doi:10.7554/eLife.100928)
Supplement: Figure 8—figure supplement 3—source data 1. [file elife-100928-fig8-figsupp3-data1.zip › Figure 8 - Figure Suppliment 3 - Source data 1/Figure Supplimentary 10 - source data 1.1 uncropped and labelled.pdf]

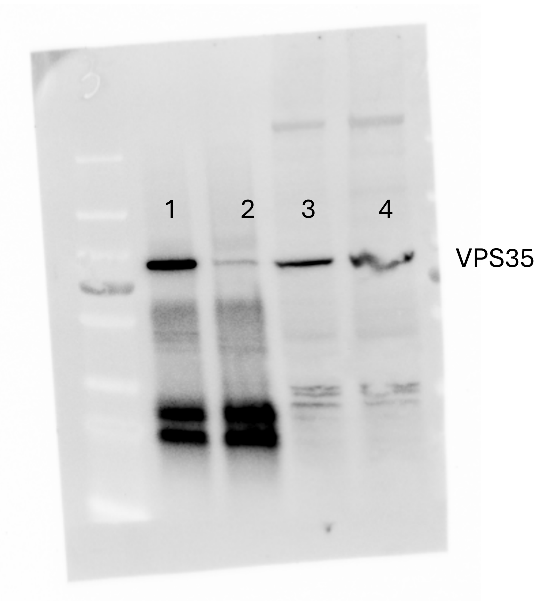

1 and 2 : CO-IP Samples
3 and 4 : Input Samples

Low Exposure

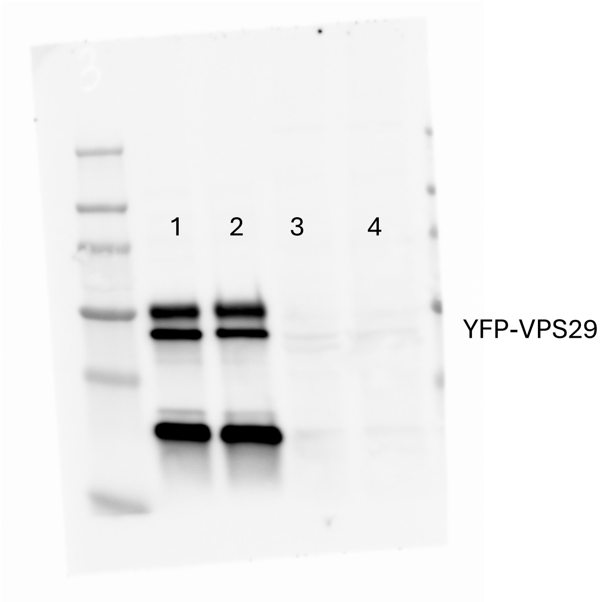

1 and 2 : CO-IP Samples
3 and 4 : Input Samples

High Exposure

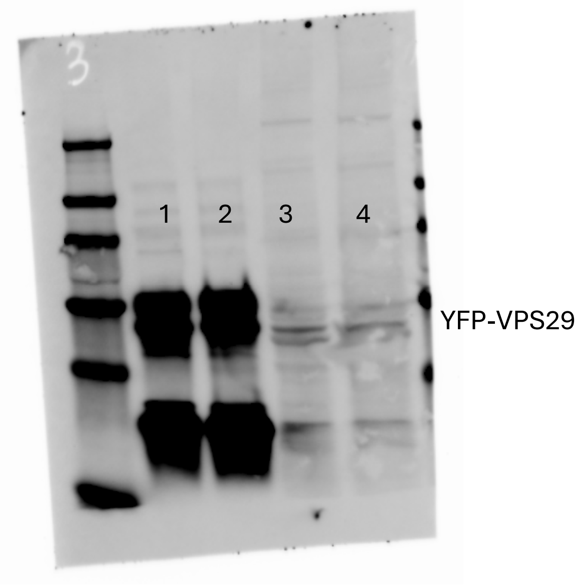

1 and 2 : CO-IP Samples
3 and 4 : Input Samples
